# Supplementary material for: Navigating the landscape of multiplayer games
Source: Nat Commun. 2020 Nov 5;11:5603. doi: 10.1038/s41467-020-19244-4 (PMC7645690; doi:10.1038/s41467-020-19244-4)
Supplement: Supplementary file 1 — Supplementary Information [file 41467_2020_19244_MOESM1_ESM.pdf]

Supplementary Information: Navigating the Landscape of Multiplayer Games

Supplementary Methods 1

This section provides additional discussions and exposition of methods.

Policy sampling scheme

The policy sampling scheme we use for large, real-world games follows the policy space coverage procedure first specified by Czarnecki et al.[22], and is detailed as follows. First, we use a tree search algorithm, Alpha-Beta[59] with varying tree depth limits d, and seeds s. This involves running the Alpha-Beta algorithm to depth d, and using random actions with seed s thereafter (i.e., if the game does not terminate). This yields policies of varying transitive strengths (controlled by depth d), with a range of related policies per depth (controlled by seed s). This also covers the case of a purely random policy when d is set to 0 (with seed s controlling the randomness). Second, we repeat the same procedure with negated game payoffs, thus also covering the space of policies that seek to lose the original game. Third, to further expand the policy space, we also define an augmented Alpha-Beta search, which assumes that branches of the game tree with depth beyond d have a value of 0. We likewise run this variant with negated payoffs as well. Finally, to cover the policy space for more difficult games, we further augment this sampling strategy by using MCTS[20] on each game, with k simulations and varying seeds s.

For each variant of Alpha-Beta detailed above, we use depth parameters $d \in \{1, \dots, 9\}$. For MCTS, we use $k \in \{10, 100, 1000\}$ simulations. For all algorithms, we also sweep over seeds $s \in \{1, \dots, 50\}$. While this sampling procedure is a heuristic, it produces a range of policies with varying degrees of transitive and intransitive relations, and thus provide a useful approximation of the underlying game.

Description of games analyzed

We provide an overview of the games analyzed (noting that we omit descriptions of 11-20, AlphaGo, MuJoCo soccer, Blotto, and AlphaStar League, as they are detailed in the main text). As our methods operate on α-Rank response graphs, they apply to many-player general-sum games. For the specific instances of two-player zero-sum games analyzed here, we symmetrize payoffs and standardize them such that $M \in [-1, 1]$.

Redundant Rock–Paper–Scissors. We modify the standard Rock–Paper–Scissors payoffs, $\mathbf{M}_{RPS}$, by duplicating the first strategy (Rock), yielding the payoffs for the redundant variant, $\mathbf{M}_{RRPS}$,

$$\mathbf{M}_{RPS} = \begin{bmatrix} 0 & -1 & 1 \\ 1 & 0 & -1 \\ -1 & 1 & 0 \end{bmatrix} \qquad \mathbf{M}_{RRPS} = \begin{bmatrix} 0 & 0 & -1 & 1 \\ 0 & 0 & -1 & 1 \\ 1 & 1 & 0 & -1 \\ -1 & -1 & 1 & 0 \end{bmatrix}. \tag{1}$$

Disc game. The Disc game is a cyclical game, defined as a differentiable generalization of the standard game of Rock–Paper–Scissors[4]. We construct the Disc game payoffs as in Czarnecki et al.[22], by first

uniformly sampling 1000 points, $\{S_i\}_{i\in[1000]}$, in the unit circle, subsequently defining payoffs,

$$M(i,j) = S_i^T \begin{bmatrix} 0 & -1 \\ 1 & 0 \end{bmatrix} S_j \,. \tag{2}$$

Elo games and noisy variants. The variety of Elo games essentially correspond to the multidimensional Elo model detailed in the main paper, with the intransitive components removed, and payoffs rescaled. Noisy variants of these Elo games are generated via specifying a noise parameter σ^2, adding zero-mean normally distributed noise of the specified variance to the non-noisy Elo payoff table, thus yielding noisy payoffs $\mathbf{M}_{\sigma^2}$. Finally, we symmetrize these payoffs, yielding final payoffs $\mathbf{M} = \mathbf{M}_{\sigma^2} - \mathbf{M}_{\sigma^2}^T$.

Games in motivating examples. The variants of transitively-structured games in the motivating examples are Elo games, as specified above. The cyclical games are $N \times N$ mElo games of rank $k = 1$, with all transitive r set to 0, and intransitive components specified as follows: $c_{0:N-2,0} = [0, 1, \ldots, N-2]$, $c_{0:N-2,1} = [N-2, N-1, \ldots, 0]$, and $c_{N-1,:} = [-1, 1]$ The random-structured games are mElo games with rank $k = 3$, and transitive and intransitive parameters i.i.d. sampled from $\mathcal{N}(0, 1)$.

Transitive game. The Transitive Game payoffs are simply set to $+1$ for the upper-triangle, -1 for the lower-triangle, and 0 across the diagonal.

Random Game of Skill. The Random Game of Skill is a 1000×1000 game, generated as defined by Czarnecki et al.[22]. Specifically, payoffs are $\mathbf{M}(i,j) = 0.5(W_{ij} - W_{ji}) + S_i - S_j$, where W_{ij}, W_{ji}, S_i, and S_j are sampled from $\mathcal{N}(0, 1)$. In the Normal Bernoulli Game, parameters S_i and S_j are sampled likewise, while W_{ij} and W_{ji} are sampled from $\mathcal{U}(0, 1)$ and payoffs are specified as $\mathbf{M}(i,j) = W_{ij} - W_{ji} + S_i - S_j$.

3-move parity game. The parity game is generated per the definition in Czarnecki et al.[22].

Real-world games. We use the OpenSpiel[50] implementations of the following games: Tic–Tac–Toe, Hex (board size=3), Quoridor (board size=3), Quoridor (board size=4), Go (board size=3), Go (board size=4), Connect Four, Kuhn Poker. For Go, we use a Komi (first-move advantage) of 6.5 points. With the exception of Kuhn Poker (which has only 12 information states, and fully enumerable policy space), we use the policy sampling scheme detailed above for this collection of games.

Supplementary Note 1: Related Work

This paper largely focuses on topological analysis and taxonomization of multiplayer games, via study of the interactions possible within them. As such, this work sits at the intersection of several different research disciplines, including game theory, machine learning, multiagent systems and network science.

A motivating application for such an approach is to classify or cluster games that present interesting challenges for artificial agents. The central question revolving around the interestingness of environments for artificial learning agents has a long history in machine learning, task theory[78], procedural content generation[80], and curriculum learning[5].

An overview of the role of games in AI, including evaluation and generation of the games themselves, is provided in Yannakakis and Togelius[93]. Thórisson et al.[78] emphasizes the need for a task theory in AI, a unifying and formal framework that enables comparison, abstraction, characterization, and decomposition of agent tasks (and the associated environments within which tasks are defined). The decompositionality of tasks enabled by such a theory can prove useful for not only understanding the task-space, but also enabling more efficient agent training via curriculum-based teaching, as discussed in Bieger and Thórisson[7]. An overview of recent AI benchmarks and platforms is provided in Hernández-Orallo et al.[40], which also includes pointers to workshops and recent works investigating the potential taxonomization of such a wide suite of tasks and environments. Overall, our contribution can be broadly classified as falling under the task theory regime, as a step towards characterizing the particular set of tasks studied here (i.e., multiplayer games that can be characterized via empirical payoff tables) via graph- and game-theoretic techniques.

Procedural generation of game content has been used in commercially-available games for decades, spanning from the dungeon-crawler and space trading games Rogue and Elite, respectively released in 1980 and 1984[9,82], to more recent expansive 3D open-world games such as No Man's Sky[31]. Significant research has also been conducted in this field, thorough overviews provided by Risi and Togelius[68], Shaker et al.[75], Togelius et al.[80]. Other notable examples of procedural content generation and automated game design include Cook and Colton[17], Cook et al.[18], Nelson and Mateas[57], Smith and Mateas[77]. The complexity of the environments generated by these and related techniques has been substantially increasing in recent years. Recently, Juliani et al.[42] introduced a procedurally generated 3D tower environment for training AI agents, which increases complexity of associated skills required by the agent to solve the task. A technique for iterative procedural task generation and AI training was introduced recently by Wang et al.[87], and further generalized in Wang et al.[88].

The literature on General Game Playing[32], in particular, has close connections to the topic studied here. Specifically, works such as Browne and Maire[10] investigate generation of complete rule-sets for extensive-form games, culminating in design of a commercially-available game (Yavalath). Related works on rule-based game generation[81] include examples generating pacman-like games[79] and chess-like games[47], with a useful survey provided in Nelson et al.[58]. A comparative evaluation of games and agents generated is provided in Perez-Liebana et al.[64].

Generation of games with attributes desirable by humans has also seen some attention, including querying of human preferences (specifically, fun, challenge, and frustration) for personalized content generation in Shaker et al.[74], creation of more believable environments[13], and creation of balanced board games[41]. Works have also taken an agent-vs.-task perspective for evaluating both agent performance and task/game quality[3,60]. A number of works have also considered specific notions of the interestingness of a game (or an evaluation of the fitness of a game from varying perspectives)[2,10,12,26,32,35,41,51,60,66,68,80,86,87,92]; we refer readers to the Landscape of Games section of the main text for discussion of these works.

In curriculum learning, initial work concerned the use of curricula of tasks in training neural networks via supervised learning[5,27,48,72]. This continues to be an active field of research to this day, with many new methods for curriculum learning being developed to match the increasingly intricate architectures used within deep learning[33,34,90], and the increasingly wide range of applications for deep neural networks, such as deep reinforcement learning[16,30,43,76,85]. Recent work has brought open-ended learning via multiagent interaction and procedural environment generation to the forefront of curriculum learning[4,22,29,43,53,87].

This work contributes to this line of inquiry by framing the intrinsic interestingness of games as a driving factor in open-ended multiagent learning. While equilibrium computation in many classes of games is PPAD-complete (thus generally considered intractable[15,23,24]), and while the computational complexity results in this work focus on the two-player zero-sum case, it is worth mentioning that our methods also directly apply to many-player general-sum settings (as exemplified by the analysis of the general-sum 11-20 game in the main text, and of many-player variants of Kuhn Poker in Supplementary Figure 10).

Several prior works have analyzed game-theoretic response graphs from the perspective of agent evaluation[1,55,56,62,70,84,89], and more generally there is a wide literature concerning analysis of directed graphs across a range of domains and disciplines[19,37,45,63]. Additionally, there is an established line of work in game theory that seeks a discrete classification of games based on payoff tables. The space of 2x2 normal-form games have been classified into groups with restrictions, such as ordering the payouts[11,54,67], and into a periodic table through topological and graph-theoretic techniques[69]. We provide a comparison against the approach of Bruns[11] below. More recent work has introduced a framework for binary classification of agent behaviors (e.g., as strategic and non-strategic) in simultaneous-move normal form games[91].

Social dilemma games (such as Prisoners Dilemma, Chicken, or Hoarding), categorized by Dawes[25], Liebrand[54], are a subset of games encapsulating the trade-offs found in social dilemmas. Social dilemmas such as the tragedy of the commons show the potential conflict between individual and group self-interest[36,61,65]. Early work in social dilemmas showed how the dilemmas can arise from reinforced behavior[65]. More recently, reinforcement learning (RL) has been applied to sequential social dilemmas[52] where strategic decisions to cooperate or defect were shown to coincide with the learning of policies. Agent behavior was also shown to depend on factors such as the relative difficulties of learning policies and in the case of cooperation, possibly non-occurring coordination sub-problems. RL has also been used to train agents that cooperate with humans across a variety of two-player repeated games[21].

In addition to analysis of individual graphs, there has been much work on the analysis and clustering of

Supplementary Table 1: 2×2 games and row player payoffs from Bruns [11]. Important note: corresponding column player payoffs are defined as a transpose along the anti-diagonal in Bruns [11] (i.e., not according to the typical convention of using standard transposes for symmetric games). This latter point has no impact on quantitative results, but is important for readers qualitatively analyzing the payoff structures and corresponding results below.

**Chicken (Ch)**	**Battle (Ba)**	**Hero (Hr)**	**Compromise (Cm)**	**Deadlock (Dl)**	**Prisoner's dilemma (Pd)**	**Stag hunt (Sh)**	**Assurance (As)**	**Coordination (Co)**	**Peace (Pc)**	**Harmony (Ha)**	**Concord (Nc)**
$\begin{bmatrix}2 & 3\\1 & 4\end{bmatrix}$	$\begin{bmatrix}3 & 2\\1 & 4\end{bmatrix}$	$\begin{bmatrix}3 & 1\\2 & 4\end{bmatrix}$	$\begin{bmatrix}2 & 1\\3 & 4\end{bmatrix}$	$\begin{bmatrix}1 & 2\\3 & 4\end{bmatrix}$	$\begin{bmatrix}1 & 3\\2 & 4\end{bmatrix}$	$\begin{bmatrix}1 & 4\\2 & 3\end{bmatrix}$	$\begin{bmatrix}1 & 4\\3 & 2\end{bmatrix}$	$\begin{bmatrix}2 & 4\\3 & 1\end{bmatrix}$	$\begin{bmatrix}3 & 4\\2 & 1\end{bmatrix}$	$\begin{bmatrix}3 & 4\\1 & 2\end{bmatrix}$	$\begin{bmatrix}2 & 4\\1 & 3\end{bmatrix}$

collections of graphs in a variety of contexts [6,46,71,83]. The graph-based analysis conducted herein draws on these works (e.g., using several local and global graph features and distributions to characterize our games, as in Berlingerio et al. [6]). However, our approach focuses specifically on games, exploiting several measures related to the complexity of games, enabling navigation of their underlying landscape and subsequent generation of new games of interest.

A wide-spread strategy comparison method in machine learning is the Elo rating [28] which ranks agents against one another in a purely transitive manner. Balduzzi et al. [3] showed the importance of intransitive strategies when comparing agents, leading to the so called multidimensional Elo model, modeling more complex relations between agents. Finally, in a line of work related to training of agents, Policy-Space Response Oracles (PSRO) [49] and Rectified PSRO [4] lead to learning a collection of possibly intransitive strategies, but not to directly analyze them, as done in this paper.

Supplementary Note 2: Additional results

This section provides additional results, including response graphs and experiments conducted for generating the landscapes in the main text.

Taxonomization of 2 × 2 games

Here we conduct a set of experiments comparing our approach against that of Bruns [11] (focusing on the ordinal games results presented in Figure 1 of their paper).

Let us first provide a summary of the approach of Bruns [11], prior to comparing our method against it. The taxonomy of 2×2 games proposed by Bruns [11] relies on two elements: 1) a topological ordering of said games, according to the patterns of payoffs received by each player; 2) classification of payoff families, based on the respective payoffs received by each player at the Nash equilibria. Based on the earlier work of Robinson and Goforth [69], Bruns [11] identifies 12 core payoff patterns in ordinal 2×2 games (which we summarize for the row player in Supplementary Table 1). Column player payoffs are defined in Bruns [11] by transposing the row player payoffs along the anti-diagonal (i.e., not the conventional transpose). The combinations of row and column player payoffs yields a total of 144 2×2 games of interest. These games are subsequently categorized according to the relative payoffs received by the players in the Nash equilibria (e.g., win-win, cyclic, unfair outcomes, etc.), yielding a total of 7 classes (reproduced here in Supplementary Figure 1a). Note that the 4 quadrants indicated by the thick blue lines in Supplementary Figure 1a indicate so-called layers of 36 games each, where games in each layer have the same highest-payoff relationship between the two players. For example, games in the lower-left layer are all win-win (thus have the highest payoff for both players in the same payoff cell), whereas games in the top-right layer have the highest payoffs in diagonally-opposite payoff cells for the players.

To generate a clustering of these games using our approach, we run the graph-based analysis detailed in the paper (i.e., computing response graphs, collecting graph measures, and running PCA on the collection of 144 games). We subsequently cluster these games using their top-2 principal components, using 7 clusters (as in Bruns [11]). The resulting clusters are visualized in Supplementary Figure 1b. Note that as our clusters are automatically discovered, and not hand-specified using Nash equilibrium payoffs, only their structure (and not color) should be compared against Bruns' clusters. While some similarities exist between these

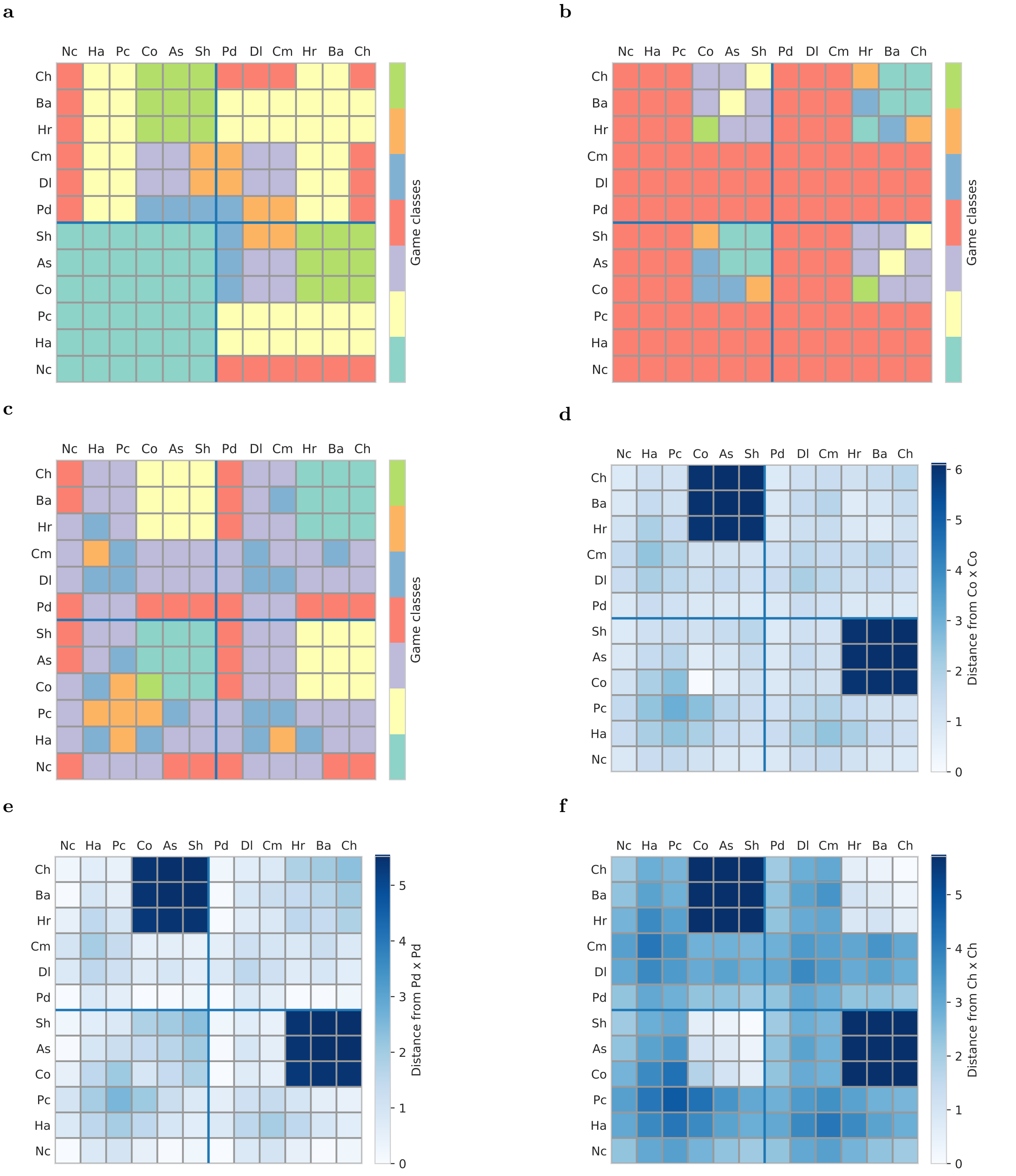

Supplementary Figure 1: Comparison of our approach to that of Bruns [11] for 2×2 games. **a** Clusters (Bruns). **b** Clusters (ours, high α-Rank α, where $\alpha = 0.2$). **c** Clusters (ours, lower α-Rank α, where $\alpha = 0.01$). **d** Game distances (ours, to $Co \times Co$). **e** Game distances (ours, to $Pd \times Pd$). **f** Game distances (ours, to $Ch \times Ch$).

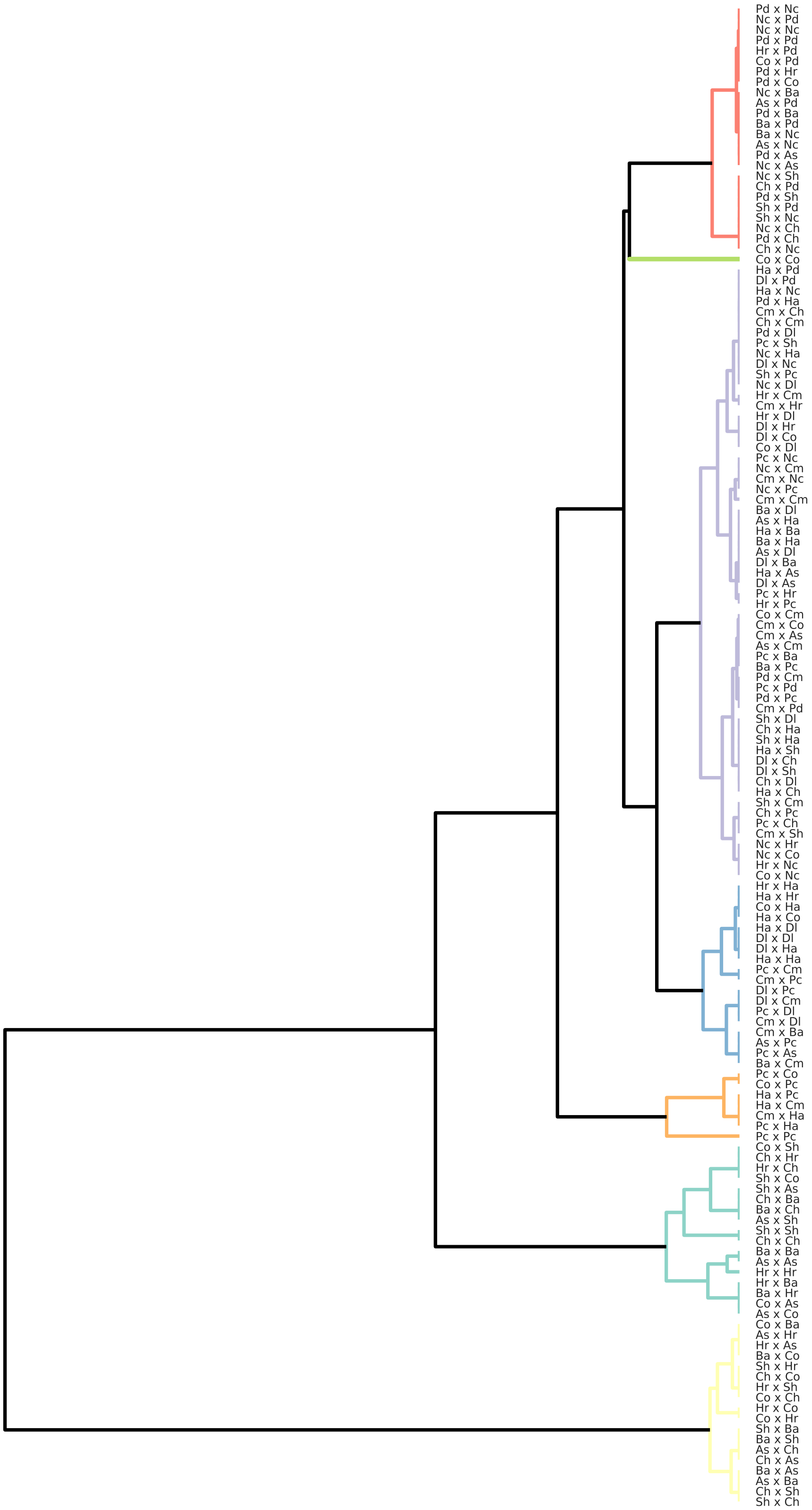

Supplementary Figure 2: Dendrogram of 2×2 games, computed using our approach. All 144 games are visualized here, with classes colored in accordance to Supplementary Figure 1c.

and Bruns' clusters (e.g., a visually-apparent division into the same 4 layers is present in our clustering, and certain games clusters such as ($Ch \times Co$, $Ch \times As$, $Ba \times Co$, $Ba \times Sh$, $Hr \times As$, $Hr \times Sh$) are shared between the two methods), notable differences are present.

To better understand these differences, consider again the means by which the Bruns clusters are derived: manual identification of patterns over the players' payoffs in the Nash equilibria of each game. Our approach, by contrast, relies on aggregation of statistics over the response graph of the games. As 2×2 games consist of 4 strategy profiles in total, the response graphs correspondingly have 4 nodes, implying that few variations are possible in their structure (in contrast to the much larger games targeted in our main results). Despite this, we can better tune our approach for these extremely small games. Specifically, one of the statistics used for computing our clustering is the α-Rank distribution entropy associated with the response graph. Typically, α-Rank is used with a high value of selection-intensity parameter, α, to increase the distribution mass over strong strategies. In these small 2×2 games, decreasing the value of α-Rank's selection-intensity parameter, α, causes the α-Rank distribution to increase in entropy, and subsequently better capture the distribution of payoffs received over the profiles (akin to the Nash-based payoff comparisons used by Bruns to derive their clusters). The results of this modification, which brings our approach closer to that of Bruns, is visualized in Supplementary Figure 1c. Notable similarities between our clusters and Bruns' are evident in this updated approach. Namely, variations of prisoner's dilemma (Pd) are clustered in our approach and theirs: ($Pd \times \{Co, As, Sh, Pd\}$ (and their transposed analogs). The cyclic game cluster of $\{Ch, Ba, Hr\} \times \{Co, As, Sh\}$ (and their transposed analogs) are identically clustered in the approaches.

Note also that due to the Nash-payoff based classification scheme of Bruns, variations in the lower-left quadrant (where all Nash equilibria are win-win) are not evident in their approach (Supplementary Table 1). By contrast, our approach highlights differences between these games. For example, whereas Bruns classifies the symmetric Coordination Game ($Co \times Co$) in the same class as Stag Hunt ($Sh \times Sh$), ours places it in its own class altogether (better matching intuition, due to the anti-coordination outcomes of Stag Hunt not being present in the coordination game).

With these comparisons in place, we further note several benefits of our approach compared to that of Bruns [11] and related works on classification of 2×2 games. First, our approach does not rely on a hand-crafted taxonomy or classification of games; our games classes are identified automatically, and without need for human expertise in determination of patterns in payoffs. Second, as the taxonomy introduced by Bruns' relies on enumeration of payoff orderings, it faces significant scalability issues if to be extended to larger games (involving more strategies and/or players). By contrast, our approach applies directly to all normal form games, and we are not aware of similar automated classification schemes for larger, many-player games. Moreover, whereas Bruns approach provides a hard classification based on hard-coded structural patterns in payoffs, ours provides a soft classification based on a similarity metric (specifically, based on the games' principal components). Thus, our approach can be used to compute distances between games, in contrast to Bruns' and related approaches. We visualize several examples of 2×2 such game distances in Supplementary Figures 1d to 1f. A subsequent benefit of being able to compute distances between games is that it enables computation of a dendrogram over a collection of games. We use our approach to visualize this tree for Bruns' 2×2 games in Supplementary Figure 2. Finally, the downstream usefulness of our approach is that such a similarity metric can be optimized to generate new games (as illustrated in the main text), in contrast to the hard classification scheme of Bruns.

Sensitivity analysis

As mentioned in the main text, the empirical game-theoretic results rely on sampling of a representative set of policies to characterize the underlying games. An important limitation here is that the the empirical game-theoretic results are subject to the policies used to generate them. Here we conduct a set of experiments to test the sensitivity of the results to these sampled policies.

For each game (with the exception of Rock–Paper–Scissors, which is a canonical game and only 3×3 in size), we randomly subsample 50% of the policies. All other policies are discarded, thus yielding an empirical payoff matrix 4 times smaller in overall size. We subsequently run the full analysis pipeline over 10 trials of subsampling per game (see Supplementary Figure 3 for results). Supplementary Figure 3a indicates the quartiles of the first two principal components per game, summarized over all trials. Despite 50% of the policies being randomly discarded, the overall statistics are relatively robust across the different trials. There

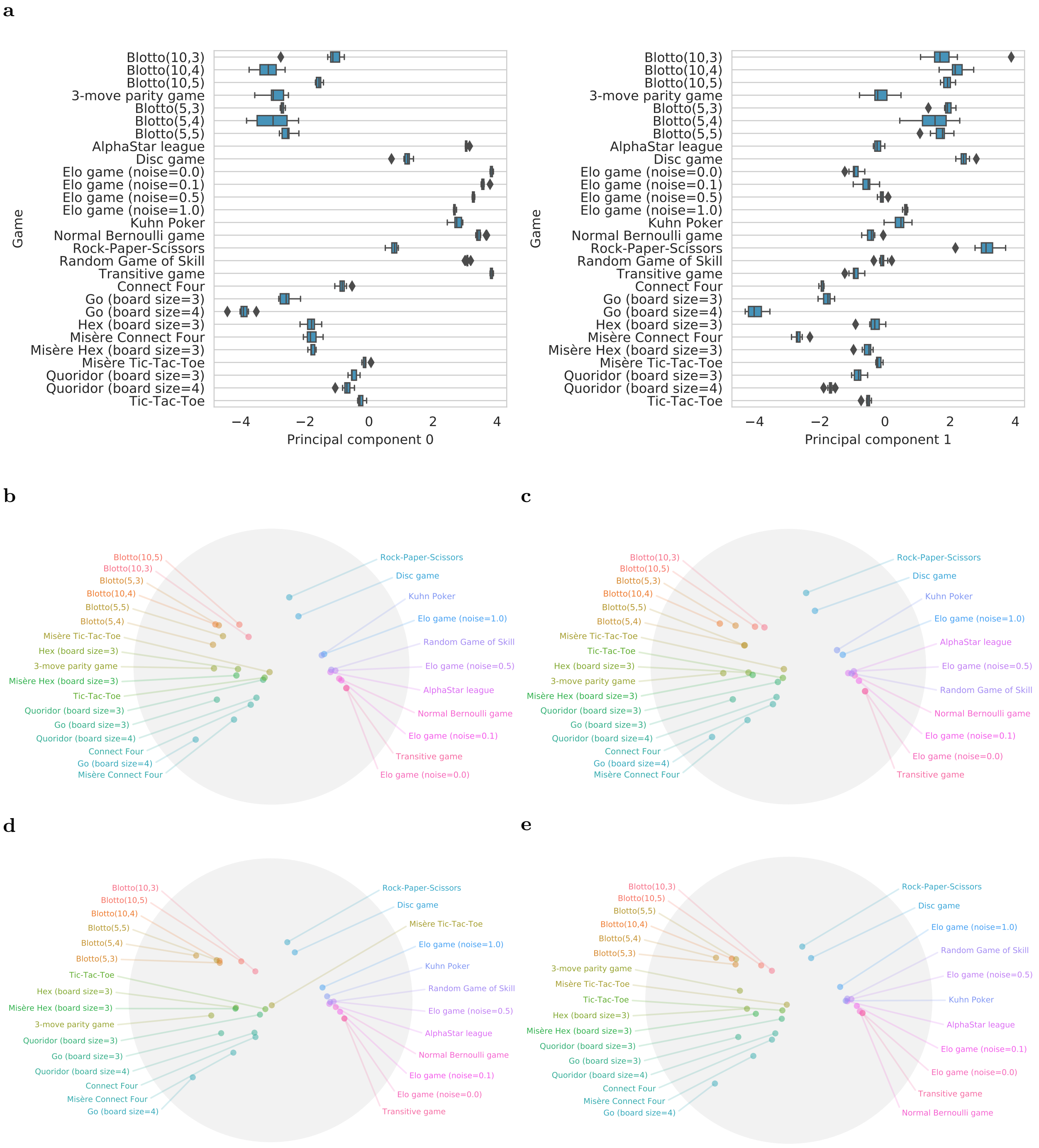

Supplementary Figure 3: Sensitivity to choice of empirical strategies, via subsampling of policy space. For each empirical game, we randomly subsample 50% of the policies (discarding the rest). We subsequently run the full analysis pipeline. 10 trials of subsampling are conducted per game (with the exception of Rock–Paper–Scissors, where we do not subsample strategies/policies due to it being a small canonical game). **a** indicates the quartiles of the first two principal components per game, over all trials. Specifically, the distribution of games' first two principal components under 10 trials of policy subsampling are illustrated, showing sensitivity to policy space changes. Boxplot elements are defined as follows: the box visualizes quartiles; whisker bars show data variability beyond the quartiles; the median is indicated by the center line; diamonds indicate outliers. **b**–**e** shows the landscape of games for 4 example trials. Note that game colors are kept the same as the original landscape of games visual (Supplementary Figure 6a) for easier comparison.

is some sensitivity of the principal components to the empirical policies sampled in the process, which on closer inspection seems to occur for highly cyclical games such as Blotto variations and the Disc game. We additionally recompute the Landscape of Games figure, independently for several of the subsampling trials, with 4 examples shown in Supplementary Figures 3b to 3e. Note that while there are some quantitative differences (in terms of relative positioning of these games in the projected space), the overall trends and clusters are quite robust to the subsampling (compared to Figure 1 of the main paper, which shows the landscape generated using all policies).

Overall, this analysis seems to indicate that the combination of our policy sampling scheme and analysis pipeline produce robust results, though as mentioned in the Discussions section of the main text, this will be a useful aspect of the method to revisit when experimenting on significantly larger games.

Impact of normalization on complexity results

We conduct additional ablative analysis of the transformations of graph complexity measures considered in the main text. As noted in the main text, these results are not intended to to propose that a specific definition of complexity (e.g., with respect to Nash) is explicitly useful for defining a topology / classification over games, but merely investigate correlations between these measures and those related to the raw response graphs. In Supplementary Figures 4 and 5, we plot all complexity results, over all combinations of normalizing/not normalizing the graph measures (y-axes) and/or the number of iterations to solve (x-axes). Note that the case corresponding to the main paper is captured by Supplementary Figure 4a, where both axes are normalized. The primary motivation for conducting this normalization (across both axes) is that the measures considered here would otherwise vary with increasing game size, which would make it easy to artificially inflate a game's complexity via arbitrary increase of the strategy space size via filler strategies.

Under this view, even the standard (i.e., non-repeated) version of Rock–Paper–Scissors has high computational complexity, in the sense that the Nash equilibrium requires these learning agents to discover and play the three pure strategies with equal probability. In other words, these results imply only that RPS is complex within the class of 3×3 games, as agents must fully explore the strategy space to equilibriate. Moreover, all larger variants of RPS be equally as complex under this view, in the sense that their Nash equilibria all have full support; thus, they would all deterministically require the same number of normalized iterations to solve (regardless of random initialization of the double oracle algorithm). Without this normalization, this particular notion of the complexity of a game could be inflated by artificially increasing the strategy space size, without affecting the underlying topology or type of strategic interactions needed to solve it.

Note that different (and potentially less useful) conclusions may indeed be drawn by considering ablations over the type of normalization done. For example, disabling this normalization yields Supplementary Figure 4b, which implies that RPS has similar complexity to a fully Transitive Game (number 17), despite the latter's Nash equilibrium being deterministically found after a single double oracle iteration. Moreover, in Supplementary Figures 4 and 5, we find that normalizing both types of measures consistently maximizes their Spearman correlation (with low p-value, see top-right of each subfigure).

We also consider now the impact that this normalization has on the landscape of games figure (see Supplementary Figure 6). Here, note that there are only 2 ablations involving only normalization over the graph measures (as double oracle/iterations to solve are not used in the spectral analysis). The normalized landscape (Supplementary Figure 6a), used in the main paper, reveals coherent clusters of related games. By contrast, the variety of underlying payoff table sizes and associated graph measures implies that the unnormalized landscape (Supplementary Figure 6b) is notably less structured. For instance, while games such as Rock–Paper–Scissors and the Disc Game are conceptually very similar, their distance is relatively larger in the unnormalized landscape due to their widely different payoff sizes (3×3 vs. 1000×1000, respectively).

Effects of mixed policies

It is interesting to consider the effect that additional mixing of policies would have on the results, and conduct a suite of experiments focused on this here. Specifically, for all games in the landscape, we expand each payoff table by adding mixtures of policies. For each game, we uniformly sample a mixture over a random

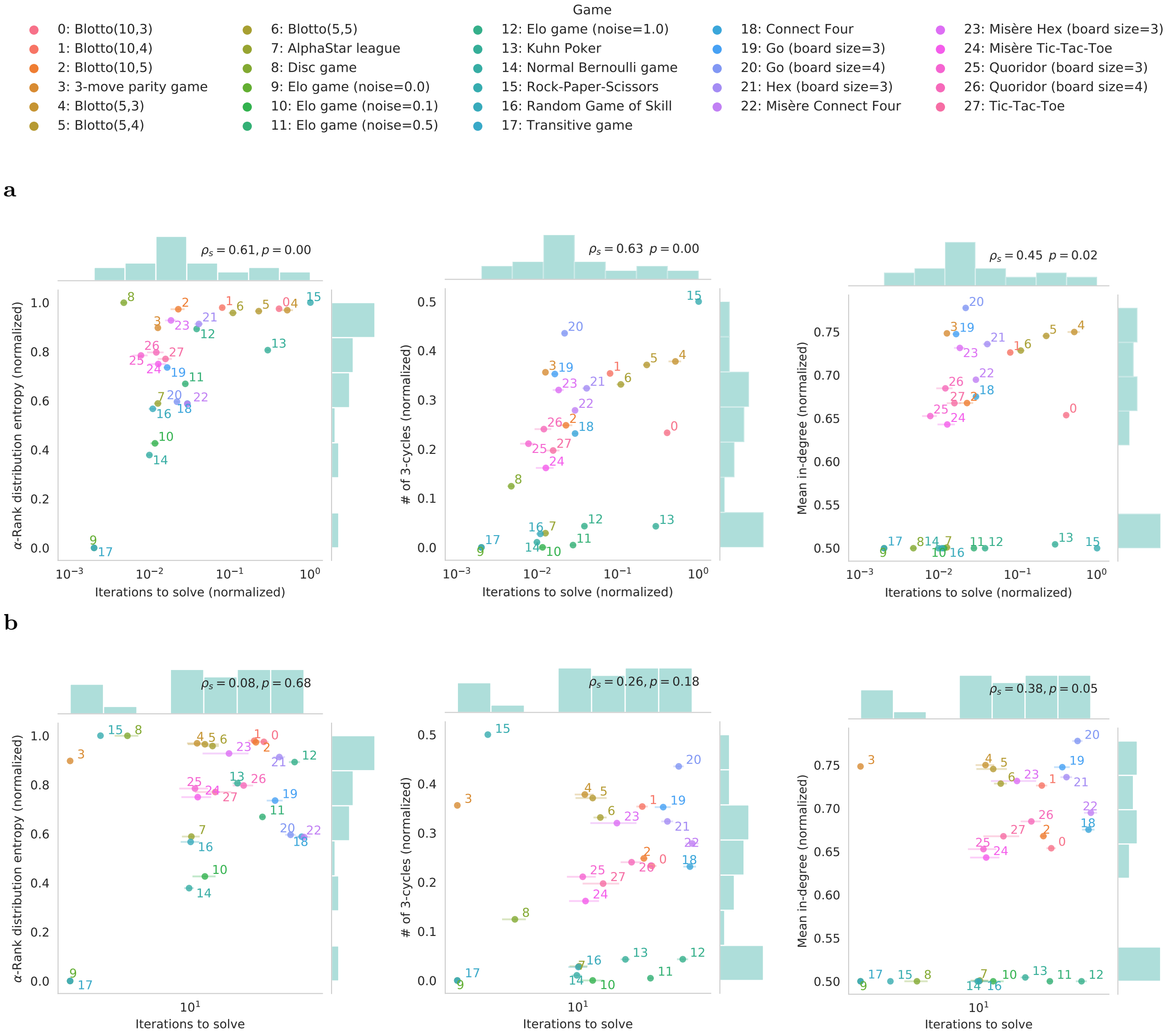

Supplementary Figure 4: Response graph complexity vs. computational complexity of solving various games. Each column plots a respective measure of graph complexity against the number of iterations needed to solve the associated game via the double oracle algorithm. In both rows, the graph complexity measures are normalized. **a** shows results where the number of double oracle iterations is also normalized. **b** shows results where the number of double oracle iterations is not normalized.

selection of half of the base policies; we repeat this 100 times, expanding the payoff table accordingly. For Rock–Paper–Scissors (due to the small size of the strategy space), we sample mixed policies uniformly over the full support of all 3 base strategies. Supplementary Figure 7 shows the results for 4 independent trials of policy mixing.

Some observations can be made here, in comparing the trials to one another (and to the original landscape, visualized in Supplementary Figure 6a). At a high-level, most of the prominent clusters found in the landscape presented in the main paper are also present here, with some specific details as follows: 1) Rock–Paper–Scissors and the Disc game (closely clustered in both, despite the size of Rock–Paper–Scissors increasing from 3 × 3 in the original landscape to 103 × 103 in the mixed policy landscapes) 2) Elo game(noise=0.0) and Transitive game; 3) Elo game(noise=0.1) and the Normal Bernoulli game; 4) Random Game of Skill, Elo game (noise=0.5), and the AlphaStar League; 5) Real-world games (e.g., Connect

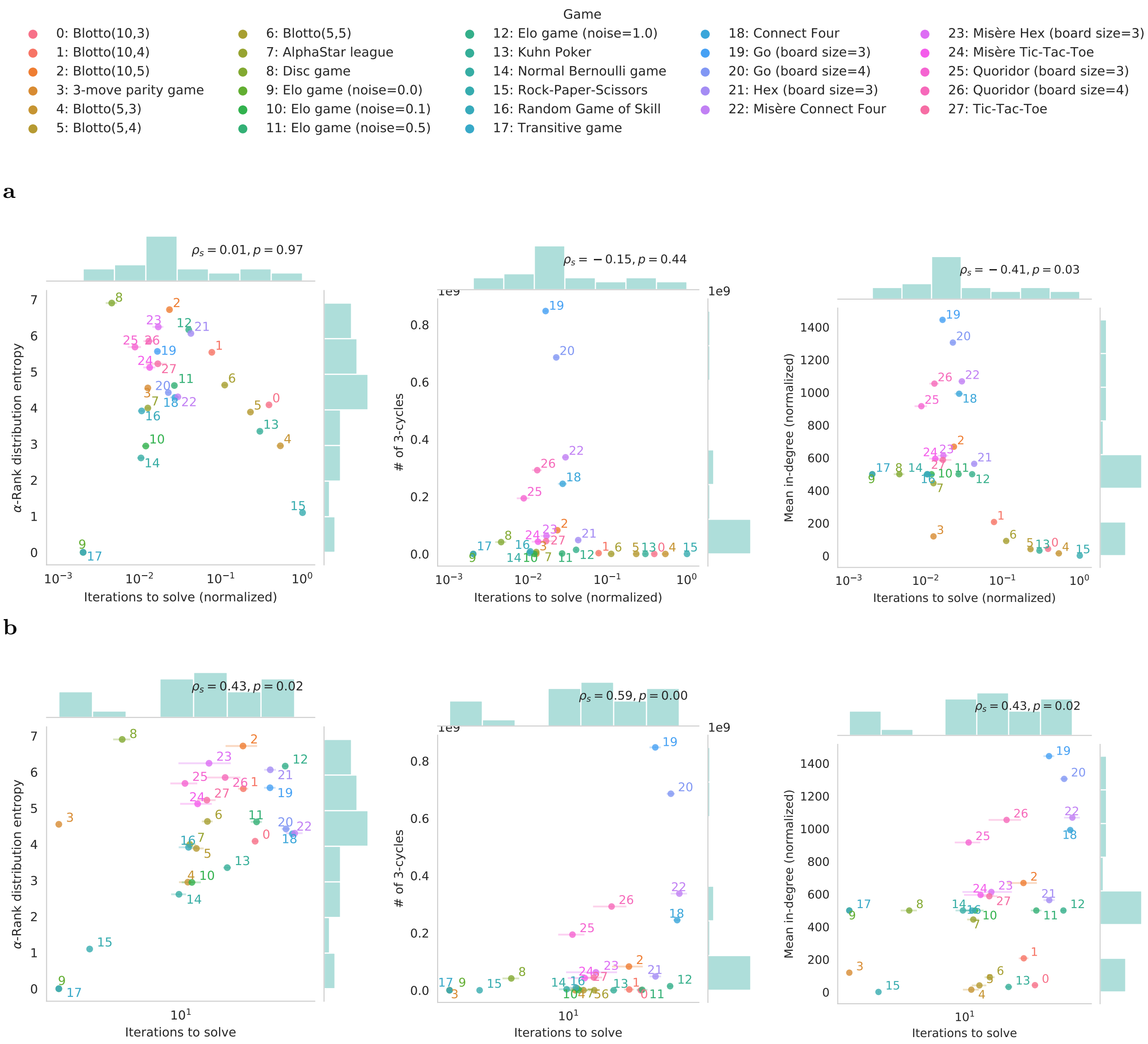

Supplementary Figure 5: Response graph complexity vs. computational complexity of solving various games. Each column plots a respective measure of graph complexity against the number of iterations needed to solve the associated game via the double oracle algorithm. In both rows, the graph complexity measures are not normalized. **a** shows results where the number of double oracle iterations is normalized. **b** shows results where the number of double oracle iterations is not normalized.

Four and Quoridor (board size=4)); 6) The cluster of Blotto games have somewhat shifted towards that of Rock–Paper–Scissors and the Disc Game. Notably, all of these games are highly cyclical, and Blotto requires players to play uniformly across all permutations of the token-selection strategies (due to the game rules itself being permutation-invariant), a characteristic shared by Rock–Paper–Scissors.

A closer look at generated games

While raw hybrids of games alone are unlikely to play a key role in establishing useful curricula, we note that discovery of core features or mechanisms in games has, indeed, been a key driver of generation of new games (see, e.g., Charity et al.[14], Khalifa et al.[44], Shaker et al.[73]). As such, we can observe interesting trends even in the specific class of normal-form games that we focus on here.

a

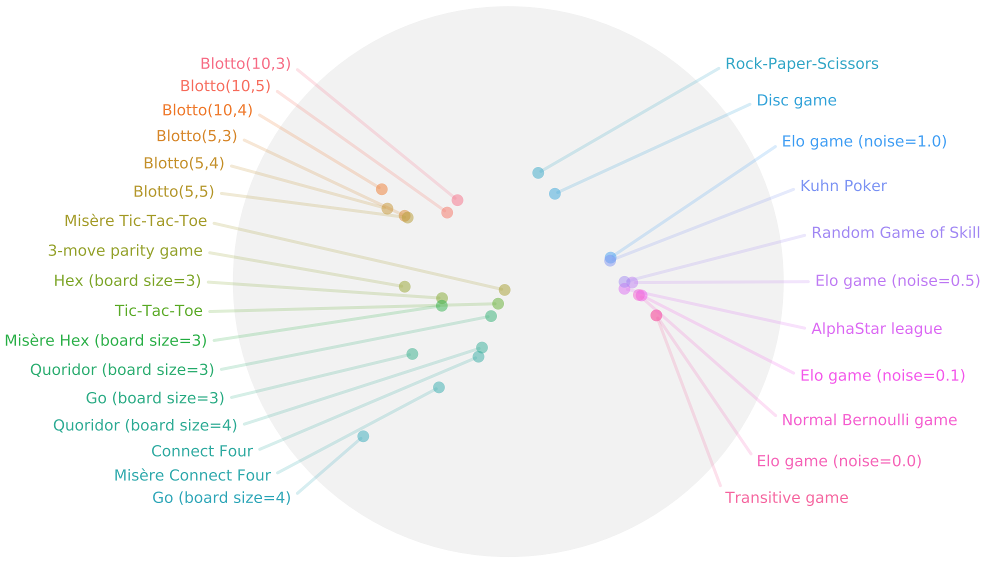

b

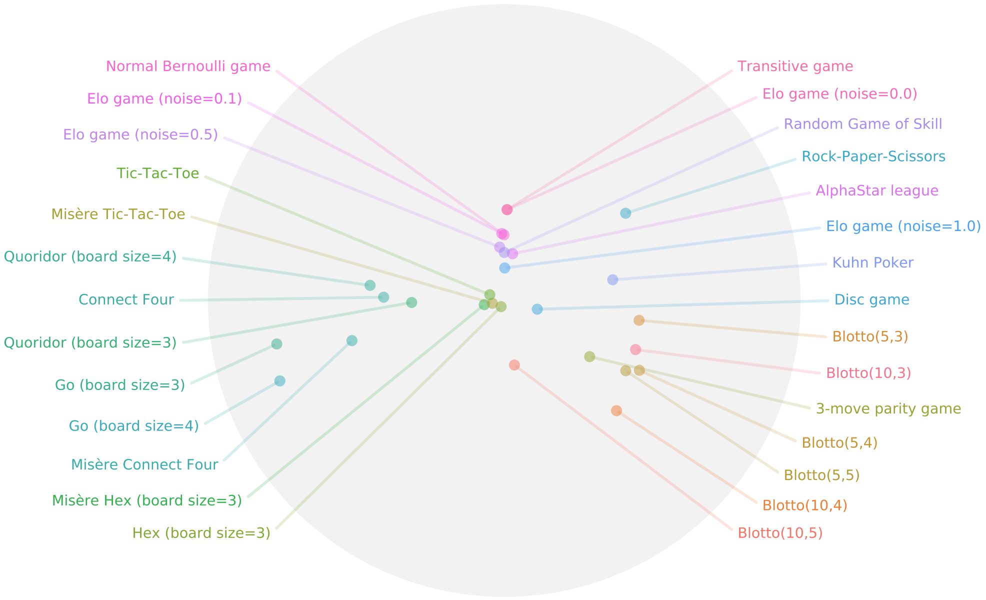

Supplementary Figure 6: Landscape of games generated via normalized and unnormalized response graph measures. **a** Normalized graph measures. **b** Unnormalized graph measures.

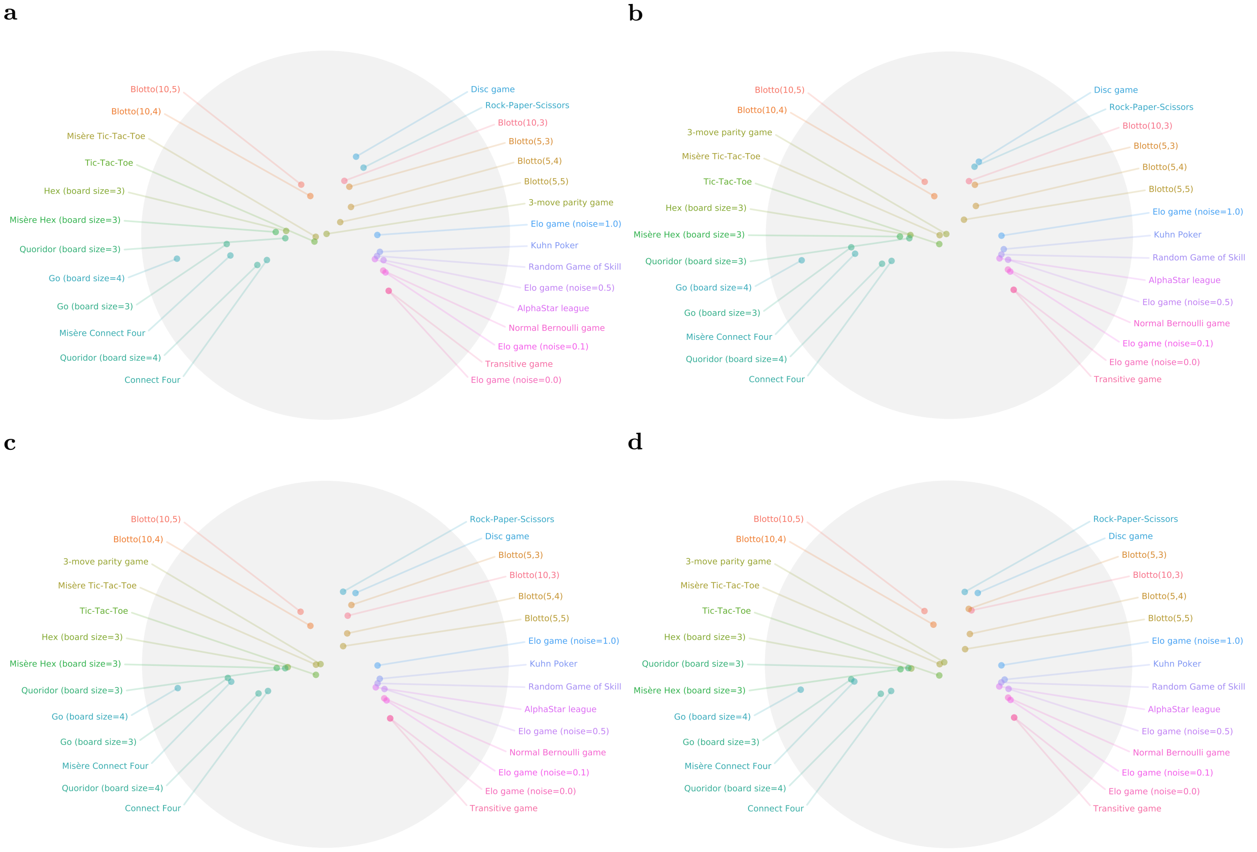

Supplementary Figure 7: Sensitivity to random mixtures of policies. In each figure, 100 additional policies are included per game, and the landscape subsequently regenerated. Note that game colors are kept the same as the original landscape of games visual (Supplementary Figure 6a) for easier comparison. **a**–**d** illustrate four such trials.

To more closely analyze trends in the games generated in the main paper, we conduct experiments that procedurally generate and subsequently train policies on these games via the double oracle algorithm. Specifically, we create a large class of generated games as follows. We first randomly sample 10 pairs of target games from the base games shown in the landscape of games (Supplementary Figure 6a). We subsequently procedurally generate games of sizes 2×2, 3×3, 5×5, 7×7, and 9×9 targeting each sampled pair. This yields a total of 50 generated games of varying characteristics. We subsequently evaluate the complexity of solving these games via double oracle (of course, these can also be used for analysis of the dynamics of multiagent learning algorithms, as done in recent works such as Bloembergen et al. [8], Hennes et al. [39]. Supplementary Figure 8 summarizes the results of this experiment, with each generated instance evaluated using 10 randomly-seeded trials of the double oracle algorithm.

We notice several trends in these generated games. First, the generated games targeting the three pairs (Blotto(10,3) $\times$ Blotto(10,4)), (Blotto(10,4) $\times$ Blotto(5,5)), and (Rock–Paper–Scissors $\times$ Disc game) consistently require the largest number of iterations to solve, across all game sizes. This result is explained by the fact that each of these games targets a mixture over pairs of highly-cyclical underlying games; thus, each generated game likely has a large Nash support (despite the noise in the generation process), making them difficult to solve. Next, we observe a lower bound roughly established by generated games involving the target pairs (Elo game (noise=0.1) $\times$ Go (board size=4)) and (Elo game (noise=0.5) $\times$ Normal Bernoulli Game). Here, the noisy Elo game plays an important role, as its (roughly) transitive structure tends to reduce the number of strong strategies, thereby making it easier to solve. Interestingly, for the largest generated game size (9×9), the pair (Transitive game $\times$ Quoridor (board size=3)) requires the fewest iterations to

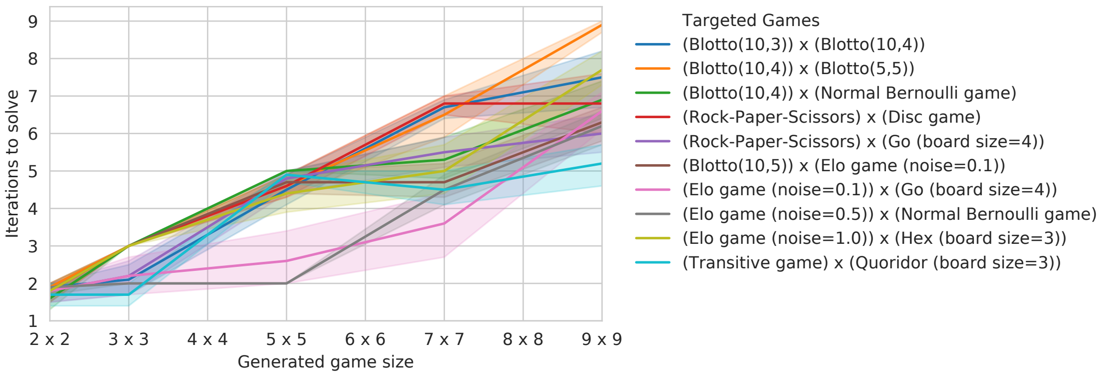

Supplementary Figure 8: Iterations to solve procedurally-generated game structures of varying sizes. Error ranges indicate 95% confidence intervals over 10 trials.

solve. Effectively, the lack of noise in the Transitive game dominates the structure of these larger generated games, in contrast to the noisy Elo games. Finally, the remaining generated games form a cluster with an intermediate number of iterations needed to solve them. This latter class of games primarily targets a mixture of cyclical and transitive games (e.g., (Blotto(10,5) $\times$ Elo game (noise=0.1)), linking to the notion of interestingness from an AI perspective, as discussed in the main text.

Overall, generation of even the simple classes of games considered here exemplifies how one might use such an analysis to highlight relationships between generated games and existing, well-studied ones, to better understand and expand the space of multiplayer games.

Motivating examples: results for randomly-generated games

Supplementary Figure 9 illustrates a sample of response graphs for generated games of random structure, as discussed in the Motivating Examples section of the main text.

Additional response graph analysis

For completeness, Supplementary Figures 10 to 15 present the response graph-based analysis for the additional games considered in the main text. Supplementary Figure 10 is of particular note here, as it exemplifies the application of the methodology to asymmetric, many-player games. Specifically, the empirical games constructed here correspond to agents trained via extensive-form fictitious play (XFP) [38] in 2-, 3-, and 4-player variants of Kuhn Poker.

Additional complexity results

Supplementary Figure 16 provides an overview of additional response graph-based measures, in comparison to the normalized number of iterations required to solve each of the games considered in the main text.

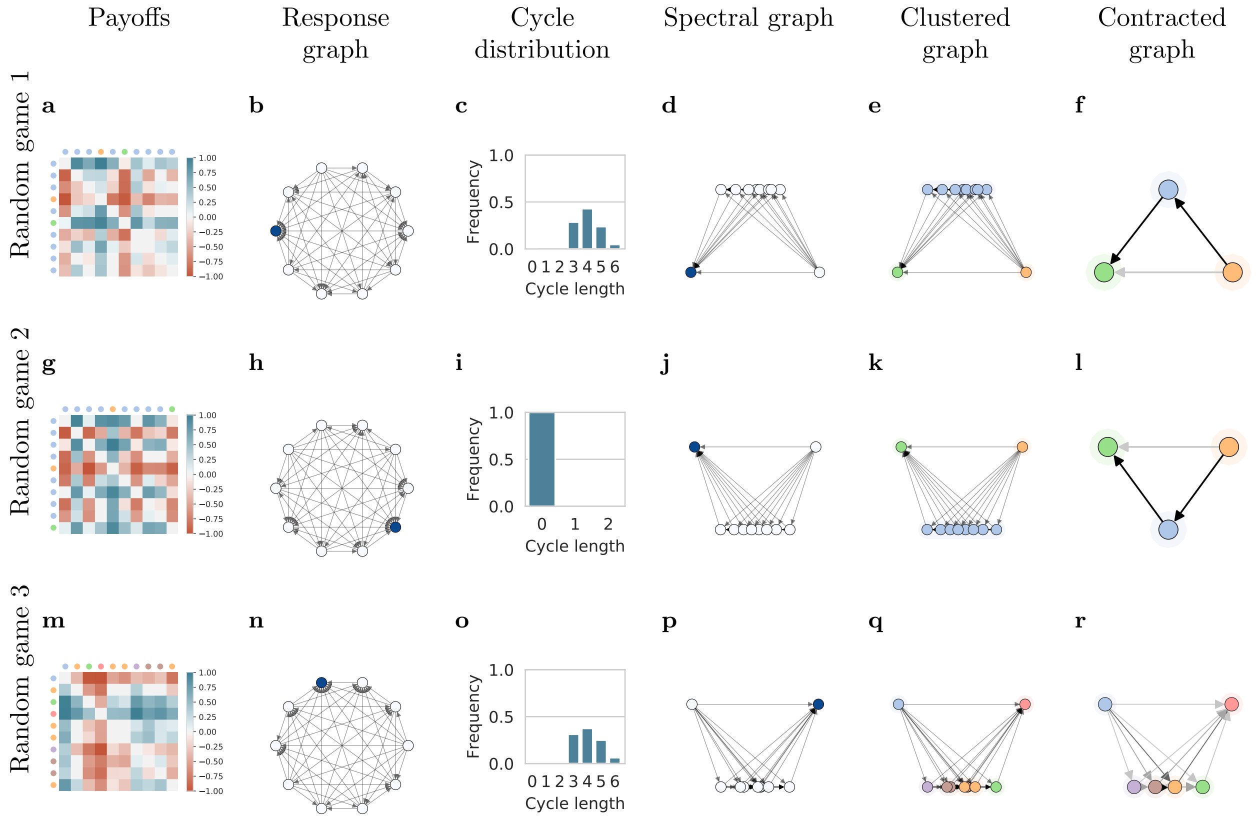

Supplementary Figure 9: Results for randomly-generated games. Each column visualizes a different characteristic of the game or response graph, as discussed in the main text.

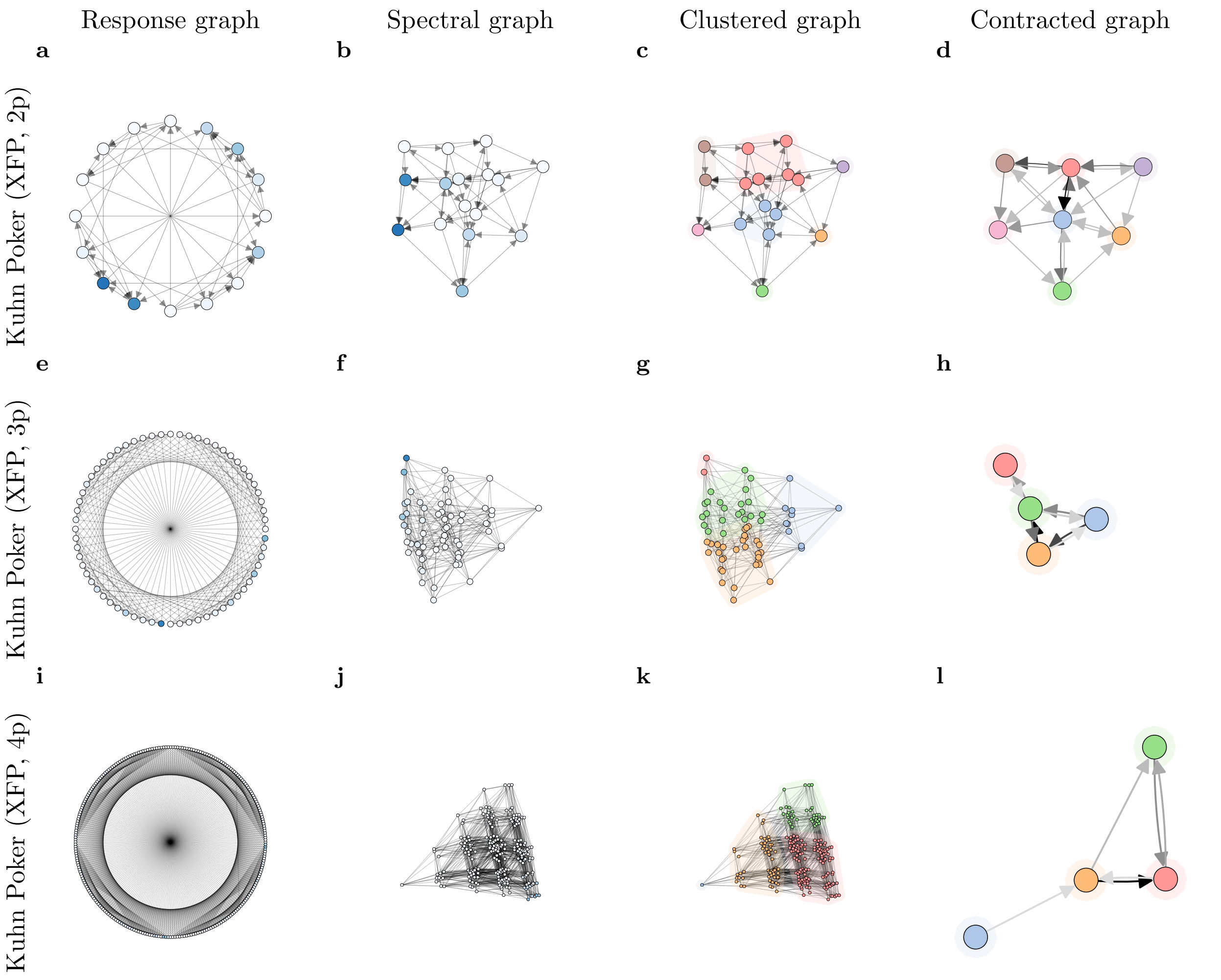

Supplementary Figure 10: Additional response graph analysis results I. Specifically, these results pertain to Kuhn Poker agents trained via extensive-form fictitious play (XFP)[38], with empirical games constructed as detailed in Omidshafiei et al.[62].

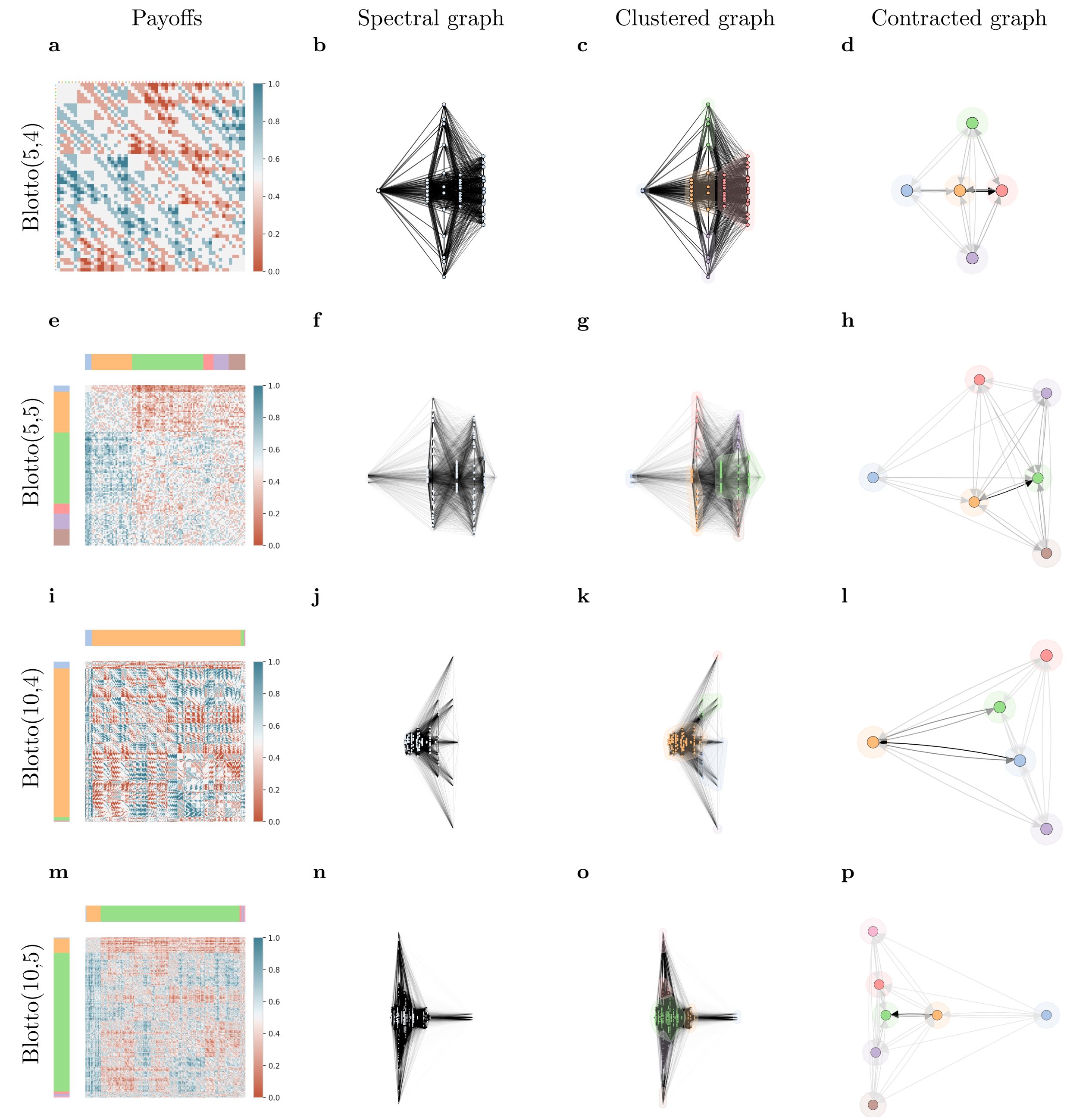

Supplementary Figure 11: Additional response graph analysis results II. Each column visualizes a different characteristic of the game or response graph, as discussed in the main text.

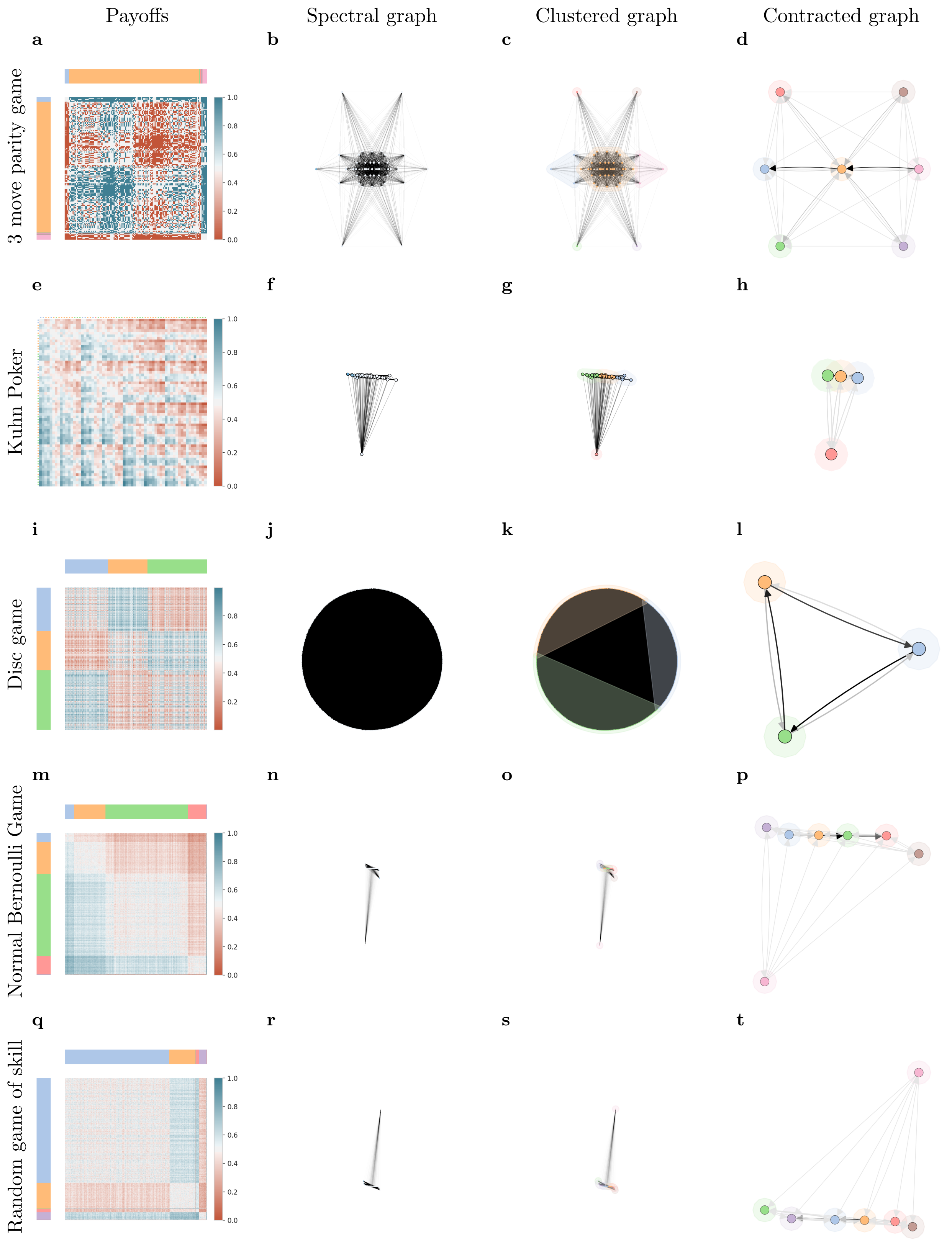

Supplementary Figure 12: Additional response graph analysis results III. Each column visualizes a different characteristic of the game or response graph, as discussed in the main text.

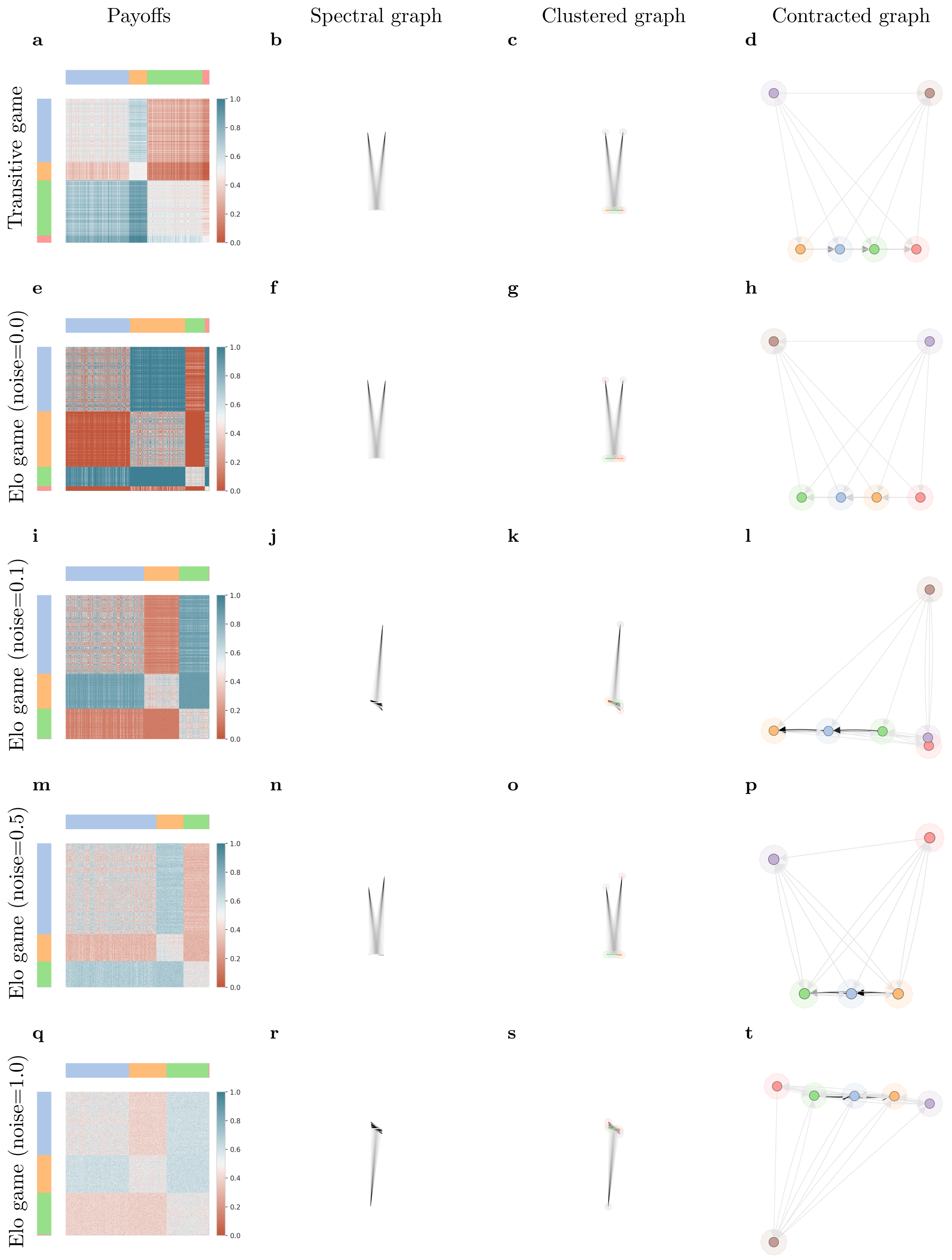

Supplementary Figure 13: Additional response graph analysis results IV. Each column visualizes a different characteristic of the game or response graph, as discussed in the main text.

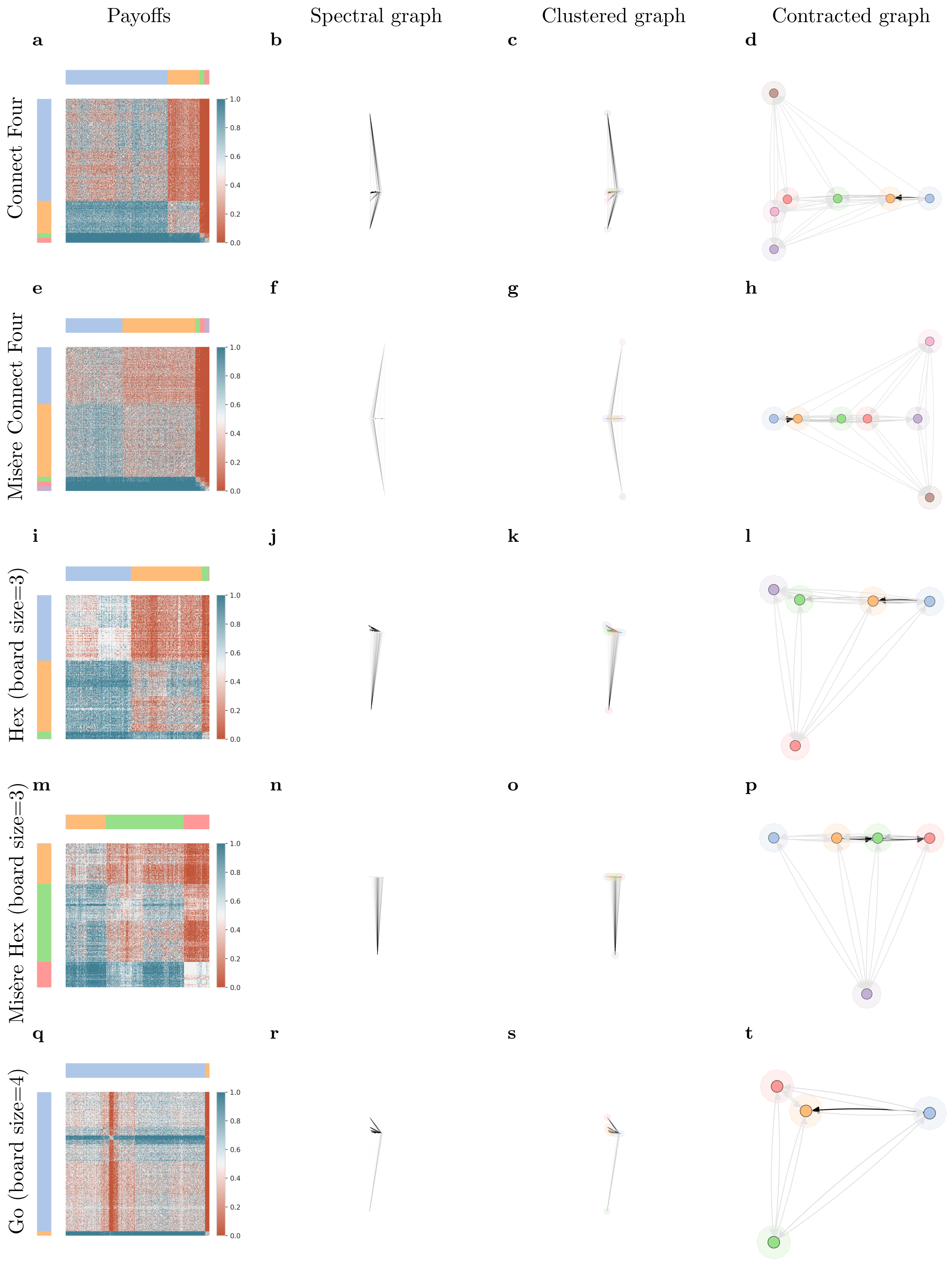

Supplementary Figure 14: Additional response graph analysis results V. Each column visualizes a different characteristic of the game or response graph, as discussed in the main text.

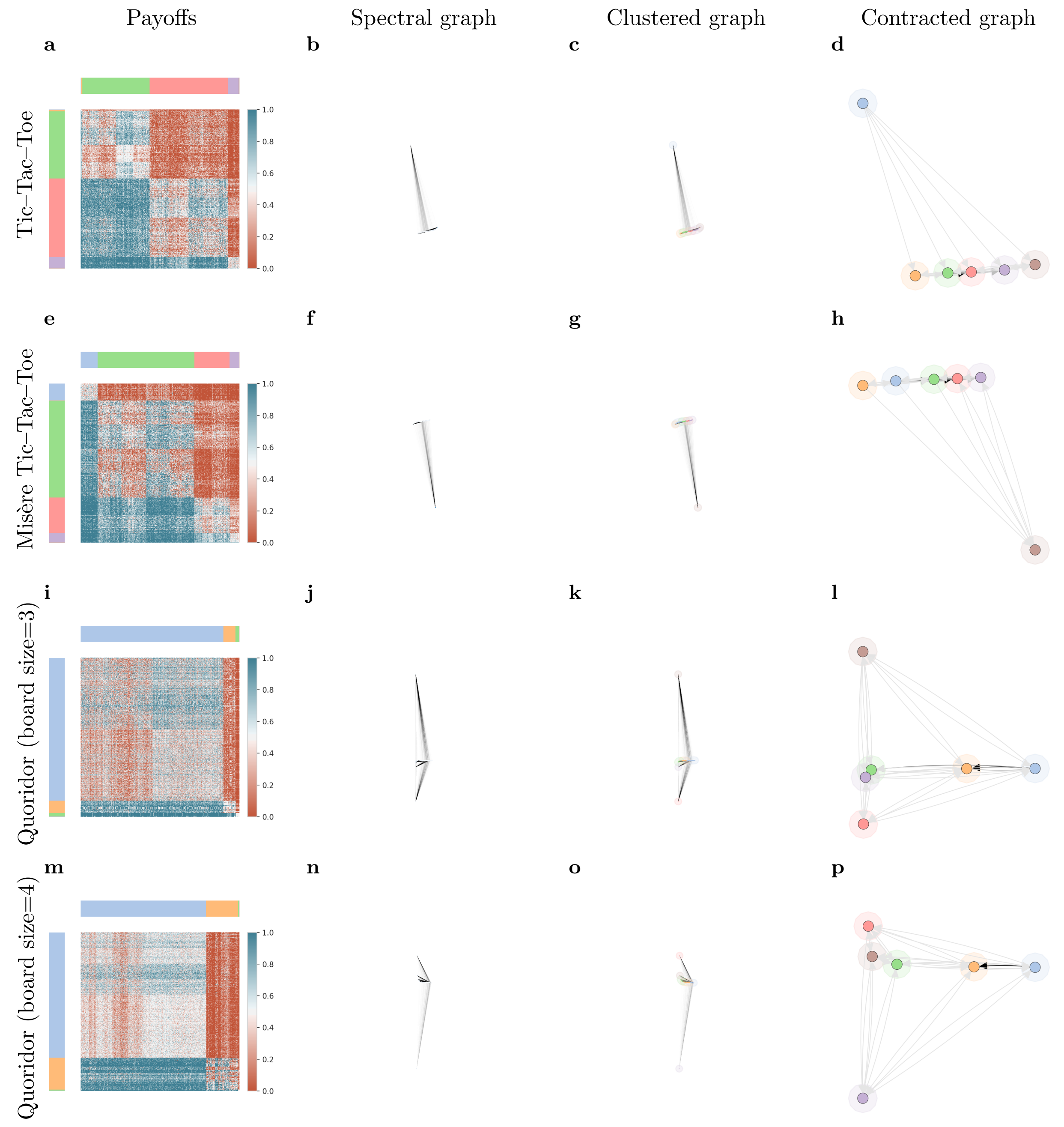

Supplementary Figure 15: Additional response graph analysis results VI. Each column visualizes a different characteristic of the game or response graph, as discussed in the main text.

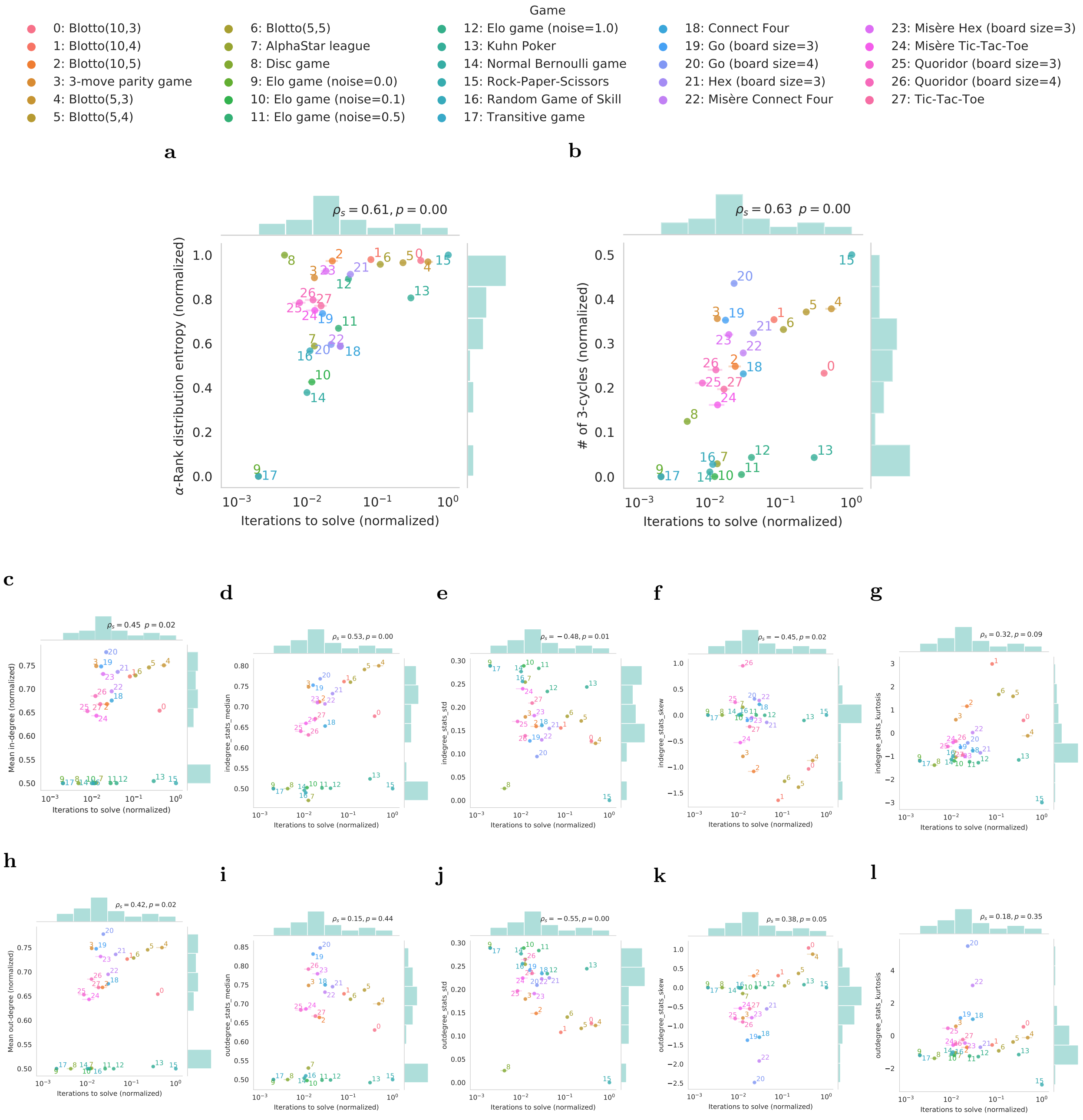

Supplementary Figure 16: Response graph complexity vs. computational complexity of solving associated games. Each figure plots a respective measure of graph complexity against the normalized number of iterations needed to solve the associated game via the double oracle algorithm (with normalization done with respect to the total number of strategies in each underlying game). Note that mean node-wise in- and out-degrees (in **c** and **h**, respectively) match across the games here due to the degree sum formula; other distributional statistics, however, do not necessarily match across in- and out-degrees, as evident above.

Supplementary References

[1] Krzysztof R Apt and Sunil Simon. A classification of weakly acyclic games. *Theo. Decis.*, 78(4):501–524, 2015.

[2] Bowen Baker, Ingmar Kanitscheider, Todor Markov, Yi Wu, Glenn Powell, Bob McGrew, and Igor Mordatch. Emergent tool use from multi-agent autocurricula. *Preprint at arXiv:1909.07528*, 2019.

[3] David Balduzzi, Karl Tuyls, Julien Perolat, and Thore Graepel. Re-evaluating evaluation. In *Proc. Neural Inf. Process. Syst.*, 2018.

[4] David Balduzzi, Marta Garnelo, Yoram Bachrach, Wojciech M Czarnecki, Julien Perolat, Max Jaderberg, and Thore Graepel. Open-ended learning in symmetric zero-sum games. In *Proc. Int. Conf. Mach. Learn.*, 2019.

[5] Yoshua Bengio, Jérôme Louradour, Ronan Collobert, and Jason Weston. Curriculum learning. In *Proc. Int. Conf. Mach. Learn.*, 2009.

[6] Michele Berlingerio, Danai Koutra, Tina Eliassi-Rad, and Christos Faloutsos. NetSimile: A scalable approach to size-independent network similarity. *Preprint at arXiv:1209.2684*, 2012.

[7] Jordi E Bieger and Kristinn R Thórisson. Task analysis for teaching cumulative learners. In *Proc. Int. Conf. on Artif Gen. Intel.*, 2018.

[8] Daan Bloembergen, Karl Tuyls, Daniel Hennes, and Michael Kaisers. Evolutionary dynamics of multi-agent learning: A survey. *J. Artif. Intel. Res.*, 53:659–697, 2015.

[9] David Braben and Ian Bell. Elite. *Firebird, Acornsoft and Imagineer*, 1984.

[10] Cameron Browne and Frederic Maire. Evolutionary game design. *IEEE T. Comp. Intel. AI*, 2(1):1–16, 2010.

[11] Bryan Randolph Bruns. Names for games: Locating 2×2 games. *Games*, 6(4):495–520, 2015.

[12] Andrew Byde. Applying evolutionary game theory to auction mechanism design. In *Proc. EEE Int. Conf. on E-Comm.* IEEE, 2003.

[13] Elizabeth Camilleri, Georgios N Yannakakis, and Alexiei Dingli. Platformer level design for player believability. In *Proc. IEEE Conf. Compu. Intel.*, 2016.

[14] Megan Charity, Michael Cerny Green, Ahmed Khalifa, and Julian Togelius. Mech-elites: Illuminating the mechanic space of gvgai. *Preprint at arXiv:2002.04733*, 2020.

[15] Xi Chen, Xiaotie Deng, and Shang-Hua Teng. Settling the complexity of computing two-player Nash equilibria. *J. ACM*, 56(3):1–57, 2009.

[16] Karl Cobbe, Oleg Klimov, Chris Hesse, Taehoon Kim, and John Schulman. Quantifying generalization in reinforcement learning. In *Proc. Int. Conf. Mach. Learn.*, 2019.

[17] Michael Cook and Simon Colton. Multi-faceted evolution of simple arcade games. In *Proc. IEEE Conf. Comp. Intel. Games*, 2011.

[18] Michael Cook, Simon Colton, and Jeremy Gow. The ANGELINA videogame design system—part I. *IEEE T. Comp. Intel. AI.*, 9(2):192–203, 2016.

[19] Luciano da F Costa, Francisco A Rodrigues, Gonzalo Travieso, and Paulino Ribeiro Villas Boas. Characterization of complex networks: A survey of measurements. *Adv. Phys.*, 56(1):167–242, 2007.

[20] Rémi Coulom. Efficient selectivity and backup operators in Monte-Carlo tree search. In *Proc. Int. Conf. Comp. Games*, 2006.

[21] Jacob W Crandall, Mayada Oudah, Fatimah Ishowo-Oloko, Sherief Abdallah, Jean-François Bonnefon, Manuel Cebrian, Azim Shariff, Michael A Goodrich, and Iyad Rahwan. Cooperating with machines. *Nat. Commun.*, 9(1):1–12, 2018.

[22] Wojciech Marian Czarnecki, Gauthier Gidel, Brendan Tracey, Karl Tuyls, Shayegan Omidshafiei, David Balduzzi, and Max Jaderberg. Real world games look like spinning tops. *Proc. Neural Inf. Process. Syst.*, 2020.

[23] Constantinos Daskalakis. On the complexity of approximating a Nash equilibrium. *ACM T. Algorithms*, 9(3):1–35, 2013.

[24] Constantinos Daskalakis, Paul W Goldberg, and Christos H Papadimitriou. The complexity of computing a Nash equilibrium. *SIAM J. on Comput.*, 39(1):195–259, 2009.

[25] Robyn M Dawes. Social dilemmas. *Annu. Rev. Psychol.*, 31(1):169–193, 1980.

[26] Sebastian Deterding. The lens of intrinsic skill atoms: A method for gameful design. *Hum.–Comput. Interact.*, 30(3-4):294–335, 2015.

[27] Jeffrey L Elman. Learning and development in neural networks: The importance of starting small. *Cognition*, 48(1):71–99, 1993.

[28] Arpad Elo. *The Rating of Chess players, Past and Present.* Ishi Press International, 1978.

[29] Richard Everett, Adam Cobb, Andrew Markham, and Stephen Roberts. Optimising worlds to evaluate and influence reinforcement learning agents. In *Proc. Auton. Agent Multi-Ag.*, 2019.

[30] Carlos Florensa, David Held, Markus Wulfmeier, Michael Zhang, and Pieter Abbeel. Reverse curriculum generation for reinforcement learning. In *Conf. on Robot Learn. (CoRL)*, 2017.

[31] Hello Games. No man's sky. *Hello Games*, 2016.

[32] Michael Genesereth, Nathaniel Love, and Barney Pell. General game playing: Overview of the AAAI competition. *AI Mag.*, 26(2):62–62, 2005.

[33] Alex Graves, Marc G Bellemare, Jacob Menick, Remi Munos, and Koray Kavukcuoglu. Automated curriculum learning for neural networks. In *Proc. Int. Conf. Mach. Learn.*, 2017.

[34] Guy Hacohen and Daphna Weinshall. On the power of curriculum learning in training deep networks. In *Proc. Int. Conf. Mach. Learn.*, 2019.

[35] Wang Hao and Sun Chuen-Tsai. Game reward systems: Gaming experiences and social meanings. In *Proc. DiGRA Int. Conf.*, 2011.

[36] Garrett Hardin. The tragedy of the commons. *Science*, 162(3859):1243–1248, 1968.

[37] Ricardo Hausmann, César A Hidalgo, Sebastián Bustos, Michele Coscia, Alexander Simoes, and Muhammed A Yildirim. *The atlas of economic complexity: Mapping paths to prosperity.* MIT Press, 2014.

[38] Johannes Heinrich, Marc Lanctot, and David Silver. Fictitious self-play in extensive-form games. In *Proc. Int. Conf. Mach. Learn.*, 2015.

[39] Daniel Hennes, Dustin Morrill, Shayegan Omidshafiei, Rémi Munos, Julien Perolat, Marc Lanctot, Audrunas Gruslys, Jean-Baptiste Lespiau, Paavo Parmas, Edgar Duéñez-Guzmán, and Karl Tuyls. Neural replicator dynamics: Multiagent learning via hedging policy gradients. In *Proc. Auton. Agent Multi-Ag.*, 2020.

[40] José Hernández-Orallo, Marco Baroni, Jordi Bieger, Nader Chmait, David L Dowe, Katja Hofmann, Fernando Martínez-Plumed, Claes Strannegård, and Kristinn R Thórisson. A new AI evaluation cosmos: Ready to play the game? *AI Mag.*, 38(3):66–69, 2017.

[41] Vincent Hom and Joe Marks. Automatic design of balanced board games. In *Proc. AAAI Conf. on Artif. Intel. and Inter. Dig. Ent.*, 2007.

[42] Arthur Juliani, Ahmed Khalifa, Vincent-Pierre Berges, Jonathan Harper, Ervin Teng, Hunter Henry, Adam Crespi, Julian Togelius, and Danny Lange. Obstacle tower: A generalization challenge in vision, control, and planning. *Preprint at arXiv:1902.01378*, 2019.

[43] Niels Justesen, Ruben Rodriguez Torrado, Philip Bontrager, Ahmed Khalifa, Julian Togelius, and Sebastian Risi. Illuminating generalization in deep reinforcement learning through procedural level generation. In *Proc. NeurIPS Deep Rein. Learn. Workshop*, 2018.

[44] Ahmed Khalifa, Michael Cerny Green, Gabriella Barros, and Julian Togelius. Intentional computational level design. In *Proc. Genetic and Evo. Comp. Conf.*, 2019.

[45] Jongkwang Kim and Thomas Wilhelm. What is a complex graph? *Physica A*, 387(11):2637–2652, 2008.

[46] Danai Koutra, Joshua T Vogelstein, and Christos Faloutsos. Deltacon: A principled massive-graph similarity function. In *IEEE Data Min.*, 2013.

[47] Jakub Kowalski and Marek Szykuła. Evolving chess-like games using relative algorithm performance profiles. In *Proc. Europ. Conf. Appl. of Evo. Comp.*, 2016.

[48] Kai A Krueger and Peter Dayan. Flexible shaping: How learning in small steps helps. *Cognition*, 110 (3):380–394, 2009.

[49] Marc Lanctot, Vinicius Zambaldi, Audrunas Gruslys, Angeliki Lazaridou, Karl Tuyls, Julien Pérolat, David Silver, and Thore Graepel. A unified game-theoretic approach to multiagent reinforcement learning. In *Proc. Neural Inf. Process. Syst.*, 2017.

[50] Marc Lanctot, Edward Lockhart, Jean-Baptiste Lespiau, Vinicius Zambaldi, Satyaki Upadhyay, Julien Pérolat, Sriram Srinivasan, Finbarr Timbers, Karl Tuyls, Shayegan Omidshafiei, Daniel Hennes, Dustin Morrill, Paul Muller, Timo Ewalds, Ryan Faulkner, János Kramár, Bart De Vylder, Brennan Saeta, James Bradbury, David Ding, Sebastian Borgeaud, Matthew Lai, Julian Schrittwieser, Thomas Anthony, Edward Hughes, Ivo Danihelka, and Jonah Ryan-Davis. OpenSpiel: A framework for reinforcement learning in games. *Preprint at arXiv:1908.09453*, 2019.

[51] Nicole Lazzaro. Why we play: affect and the fun of games. *Hum.–Comput. Interact.*, 155:679–700, 2009.

[52] Joel Z Leibo, Vinicius Zambaldi, Marc Lanctot, Janusz Marecki, and Thore Graepel. Multi-agent reinforcement learning in sequential social dilemmas. In *Proc. Auton. Agent Multi-Ag.*, 2017.

[53] Joel Z Leibo, Edward Hughes, Marc Lanctot, and Thore Graepel. Autocurricula and the emergence of innovation from social interaction: A manifesto for multi-agent intelligence research. *Preprint at arXiv:1903.00742*, 2019.

[54] Wim BG Liebrand. A classification of social dilemma games. *Sim. & Games*, 14(2):123–138, 1983.

[55] Vahab S Mirrokni and Adrian Vetta. Convergence issues in competitive games. In *Approximation, randomization, and combinatorial optimization: Algorithms and techniques.* Springer, 2004.

[56] Paul Muller, Shayegan Omidshafiei, Mark Rowland, Karl Tuyls, Julien Perolat, Siqi Liu, Daniel Hennes, Luke Marris, Marc Lanctot, Edward Hughes, Zhe Wang, Guy Lever, Nicolas Heess, Thore Graepel, and Remi Munos. A generalized training approach for multiagent learning. In *Proc. Int. Conf. Learn. Represent*, 2020.

[57] Mark J Nelson and Michael Mateas. Towards automated game design. In *Proc. Cong. Ital. Assoc. Artif. Intel.*, 2007.

[58] Mark J Nelson, Julian Togelius, Cameron Browne, and Michael Cook. Rules and mechanics. In *Proc. Proced. Content Gen. Games*, pages 99–121. Springer, 2016.

[59] Allen Newell and Herbert A Simon. Computer science as empirical inquiry: Symbols and search. *Commun. ACM*, 1976.

[60] Thorbjørn S Nielsen, Gabriella AB Barros, Julian Togelius, and Mark J Nelson. Towards generating arcade game rules with VGDL. In *Proc. IEEE Conf. Compu. Intel.*, 2015.

[61] Mancur Olson. *The Logic of Collective Action.* Harvard University Press, 1974.

[62] Shayegan Omidshafiei, Christos Papadimitriou, Georgios Piliouras, Karl Tuyls, Mark Rowland, Jean-Baptiste Lespiau, Wojciech M Czarnecki, Marc Lanctot, Julien Perolat, and Rémi Munos. α-Rank: Multi-agent evaluation by evolution. *Sci. Rep.*, 9, 2019.

[63] Lawrence Page, Sergey Brin, Rajeev Motwani, and Terry Winograd. The PageRank citation ranking: Bringing order to the web. Technical report, Stanford InfoLab, 1999.

[64] Diego Perez-Liebana, Jialin Liu, Ahmed Khalifa, Raluca D Gaina, Julian Togelius, and Simon M Lucas. General video game AI: A multitrack framework for evaluating agents, games, and content generation algorithms. *IEEE Trans. Games*, 11(3):195–214, 2019.

[65] John Platt. Social traps. *Am. Psychol.*, 28(8):641, 1973.

[66] Marc Prensky. Fun, play and games: What makes games engaging. *Dig. Game-based Learn.*, 5(1):5–31, 2001.

[67] Anatol Rapoport and Melvin Guyer. A taxonomy of 2×2 games. *Gen. Sys.*, 11:203–214, 1966.

[68] Sebastian Risi and Julian Togelius. Increasing generality in machine learning through procedural content generation. *Nature Mach. Intel.*, pages 1–9, 2020.

[69] David Robinson and David Goforth. *The topology of the 2x2 games: A new periodic table*, volume 3. Psychology Press, 2005.

[70] Mark Rowland, Shayegan Omidshafiei, Karl Tuyls, Julien Perolat, Michal Valko, Georgios Piliouras, and Remi Munos. Multiagent evaluation under incomplete information. In *Proc. Neural Inf. Process. Syst.*, 2019.

[71] A Sanfeliu and King-Sun Fu. A distance measure between attributed relational graphs for pattern recognition. *IEEE T. Sys., Man, Cyber.*, SMC-13(3), 1983.

[72] Terence D Sanger. Neural network learning control of robot manipulators using gradually increasing task difficulty. *IEEE T. Robotic Autom.*, 10(3):323–333, 1994.

[73] Mohammad Shaker, Mhd Hasan Sarhan, Ola Al Naameh, Noor Shaker, and Julian Togelius. Automatic generation and analysis of physics-based puzzle games. In *Proc. IEEE Conf. Compu. Intel.*, 2013.

[74] Noor Shaker, Georgios Yannakakis, and Julian Togelius. Towards automatic personalized content generation for platform games. In *Proc. Artif. Intel. and Int. Dig. Ent. Conf.*, 2010.

[75] Noor Shaker, Julian Togelius, and Mark J Nelson. *Procedural content generation in games.* Springer, 2016.

[76] David Silver, Thomas Hubert, Julian Schrittwieser, Ioannis Antonoglou, Matthew Lai, Arthur Guez, Marc Lanctot, Laurent Sifre, Dharshan Kumaran, Thore Graepel, Timothy Lillicrap, Karen Simonyan, and Demis Hassabis. A general reinforcement learning algorithm that masters chess, shogi, and Go through self-play. *Science*, 362(6419):1140–1144, 2018.

[77] Adam M Smith and Michael Mateas. Answer set programming for procedural content generation: A design space approach. *IEEE T. Comp. Intel. AI.*, 3(3):187–200, 2011.

[78] Kristinn R Thórisson, Jordi Bieger, Thröstur Thorarensen, Jóna S Siguroardóttir, and Bas R Steunebrink. Why artificial intelligence needs a task theory. In *Proc. Int. Conf. on Artif Gen. Intel.*, 2016.

[79] Julian Togelius and Jurgen Schmidhuber. An experiment in automatic game design. In *Proc. IEEE Conf. Compu. Intel.*, 2008.

[80] Julian Togelius, Georgios N Yannakakis, Kenneth O Stanley, and Cameron Browne. Search-based procedural content generation: A taxonomy and survey. *IEEE T. Comp. Intel. AI*, 3(3):172–186, 2011.

[81] Julian Togelius, Mark Nelson, and Antonios Liapis. Characteristics of generatable games. In *Work. Proced. Content Gen. Games*, 2014.

[82] Michael Toy and Glenn Wichman. Rogue. Cross-platform, 1980.

[83] Anton Tsitsulin, Davide Mottin, Panagiotis Karras, Alexander Bronstein, and Emmanuel Müller. NetLSD: Hearing the shape of a graph. In *Proc. Int. Conf. on Know. Disc. & Data Min. (SIGKDD)*, 2018.

[84] Valeriu Ungureanu and Ana Botnari. Nash equilibria sets in mixed extended 2×3 games. *Comp. Sci. J. Mold.*, 13(2):38, 2005.

[85] Oriol Vinyals, Igor Babuschkin, Wojciech M. Czarnecki, Michaël Mathieu, Andrew Dudzik, Junyoung Chung, David H. Choi, Richard Powell, Timo Ewalds, Petko Georgiev, Junhyuk Oh, Dan Horgan, Manuel Kroiss, Ivo Danihelka, Aja Huang, Laurent Sifre, Trevor Cai, John P. Agapiou, Max Jaderberg, Alexander S. Vezhnevets, Rémi Leblond, Tobias Pohlen, Valentin Dalibard, David Budden, Yury Sulsky, James Molloy, Tom L. Paine, Caglar Gulcehre, Ziyu Wang, Tobias Pfaff, Yuhuai Wu, Roman Ring, Dani Yogatama, Dario Wünsch, Katrina McKinney, Oliver Smith, Tom Schaul, Timothy Lillicrap, Koray Kavukcuoglu, Demis Hassabis, Chris Apps, and David Silver. Grandmaster level in StarCraft II using multi-agent reinforcement learning. *Nature*, 575(7782):350–354, 2019.

[86] Lev Vygotsky. Interaction between learning and development. *Read. Dev. Child.*, 23(3):34–41, 1978.

[87] Rui Wang, Joel Lehman, Jeff Clune, and Kenneth O Stanley. Paired open-ended trailblazer (POET): Endlessly generating increasingly complex and diverse learning environments and their solutions. *Preprint at arXiv:1901.01753*, 2019.

[88] Rui Wang, Joel Lehman, Aditya Rawal, Jiale Zhi, Yulun Li, Jeff Clune, and Kenneth O Stanley. Enhanced POET: Open-ended reinforcement learning through unbounded invention of learning challenges and their solutions. *Preprint at arXiv:2003.08536*, 2020.

[89] Xiaofeng Wang and Tuomas Sandholm. Reinforcement learning to play an optimal Nash equilibrium in team Markov games. In *Proc. Neural Inf. Process. Syst.*, 2003.

[90] Daphna Weinshall, Gad Cohen, and Dan Amir. Curriculum learning by transfer learning: Theory and experiments with deep networks. In *Proc. Int. Conf. Mach. Learn.*, 2018.

[91] James R Wright and Kevin Leyton-Brown. A formal separation between strategic and nonstrategic behavior. *Preprint at arXiv:1812.11571*, 2018.

[92] Georgios N Yannakakis and John Hallam. Evolving opponents for interesting interactive computer games. In *Proc. Int. Conf. on Sim. Adapt. Beh.*, 2004.

[93] Georgios N Yannakakis and Julian Togelius. *Artificial intelligence and games.* Springer, 2018.
